# Effectiveness of an interactive web-based health program for adults: a study protocol for three concurrent controlled-randomized trials (EVA-TK-Coach)

**DOI:** 10.1186/s13063-021-05470-8

**Published:** 2021-08-10

**Authors:** Iris Tinsel, Gloria Metzner, Christian Schlett, Matthias Sehlbrede, Martina Bischoff, Robin Anger, Judith Brame, Daniel König, Ramona Wurst, Reinhard Fuchs, Peter Lindinger, Rainer Bredenkamp, Erik Farin-Glattacker

**Affiliations:** 1 Section of Health Care Research and Rehabilitation Research (SEVERA), Medical Center – University of Freiburg, Faculty of Medicine, University of Freiburg, Hugstetter Straße 49, 79106 Freiburg, Germany; 2 Department of Sport and Sport Science (IfSS), University of Freiburg, Freiburg, Germany; 3 Scientific Working Group in Smoking Cessation (WAT), Tübingen, Germany; 4 Clinical Trials Unit UMG, University Medical Center Göttingen, Georg-August-University, Göttingen, Germany

**Keywords:** Primary prevention, Health behavior, Healthy lifestyle, Smoking cessation, Physical fitness, Weight loss, Randomized controlled trial, Internet based intervention, Web-based health program, Behavior change

## Abstract

**Background:**

A healthy lifestyle can help prevent diseases that impair quality of life and lead to premature death. The *Techniker health insurance fund* offers a comprehensive online health program to support users in achieving their health goals of *Increasing Fitness*, *Losing and Maintaining Weight*, or *Smoking Cessation*.

**Methods:**

The aim of this study is to test the long-term effectiveness of the web-based *TK-HealthCoach* with regard to the primary outcomes of increased physical activity, sustainable weight reduction, and smoking abstinence. We are conducting three interconnected, randomized controlled trials (RCT), one for each health goal, within which participants are allocated to an intervention group (interactive online health program) or a control group (non-interactive online health program).

The effects of the intervention groups compared to the control groups will be analyzed by multi-level models for change. Participants’ data are captured via online questionnaires before the program starts (baseline t0), again when it ends (t1), and later at two follow-up surveys (t2 and t3); the latter 12 months after t1. We are documenting socio-demographic, health-related, and psychological variables as well as usage behavior data of the programs.

According to our sample size calculation, we have to enroll 1114 participants in each *Losing and Maintaining Weight* and *Increasing Fitness* RCT and 339 participants in the *Smoking Cessation* RCT. Additionally, 15–20 participants in the interactive smoking-cessation program will be invited to qualitative telephone interviews with the aim to obtain detailed information concerning utilization, compliance, and satisfaction.

The online RCTs’ inclusion criteria are: adults of each gender regardless of whether they are insured with *Techniker health insurance fund*. Persons with impairments or pre-existing conditions require a medical assessment as to whether the program is suitable for them. Specific exclusion criteria apply to each program/RCT.

**Discussion:**

We assume that study participants will improve their health behavior by using the offered online health programs and that each health goal’s intervention group will reveal advantages regarding the outcome variables compared to the control groups. Study enrollment started on January 1, 2020.

**Trial registration:**

German Clinical Trials Register, Universal Trial Number (UTN): U1111-1245-0273. Registered on 11 December 2019

**Supplementary Information:**

The online version contains supplementary material available at 10.1186/s13063-021-05470-8.

## Background

Consumer habits and working conditions strongly influence each member of society’s lifestyle. Smoking, low physical activity and obesity lead to increased individual health risks, e.g., for cardiovascular diseases, cancer, diabetes mellitus, musculoskeletal diseases, etc., putting a very high financial burden on the healthcare system. Recommendations have been made globally and programs developed to support a healthy lifestyle [1–3]. The earlier a healthy lifestyle is implemented, the less likely one is to suffer from the consequences of tobacco consumption, low physical activity, or obesity. However, lifestyle changes are particularly hard to realize when disease-related burdens are not yet apparent. Moreover, people free of cardiovascular diseases but carrying risks like smoking, hypertension, diabetes mellitus, and obesity who initially took statins or antihypertensive drugs decreased physical activity and increased weight, as Korhonen et al. found in their cohort study with > 41,000 participants [4].

Digital programs promoting primary prevention can play an important role for working-age adults. There are already many commercial and non-commercial online programs to support preventive health behavior, although the variety on offer is immense, as is their quality [5]. Some online programs were tested in randomized-controlled trials, but as their content, duration, and possibilities of interactivity differed, the effectiveness varied widely. However, results show that the more individualized and interactive online interventions are aligned, the stronger are their effects [6–13]. Nevertheless, we must keep in mind that strong interactivity and close ties to a web-based program may also make participants feel overly obliged, which may cause users to quit the program prematurely. Particular motivational and volitional factors for changing health behavior are therefore very important [14, 15]. The content of an E-Coach, its individual usability and degree of interaction, and its technical functionality and design are playing an important role to encourage user’s program adherence and thus a successful change in behavior [14].

Digital health services are often highly commercialized, and there is a myriad of health programs of very different quality offered by commercial and non-commercial institutions and health insurers. It is difficult for users to assess the effectiveness and security of the programs. Ineffective health programs are not only unnecessarily costly—they may also cause harm or demotivate participants sustainably.

It is therefore necessary to ensure that such digital applications make their benefits and effectiveness transparent to potential users [16–18]. Furthermore, scientific and comparable quality criteria should be developed to support both customers and health care providers choose or offer effective digital health programs.

To improve disease prevention, the German *Techniker (TK) health insurance fund* developed, with support from experts in medicine, sports science, psychology, and motivational psychology, a comprehensive online health coach to encourage users to achieve individual health goals. Three of these health goals—*Increasing Fitness*, *Losing and Maintaining Weight* and *Smoking Cessation*—will be independently examined by the Section of Health Care Research and Rehabilitation Research (SEVERA) and Medical Center Freiburg and the Department of Sport and Sport Science (IfSS)—both located in the University of Freiburg.

SEVERA is mainly responsible for coordinating the overall study and its summative evaluation, which is being carried out via three online randomized controlled trials (RCTs) (one for each health goal). It is also responsible for the formative evaluation of the intervention and control group programs with the *Smoking Cessation* health goal.

The IfSS is responsible for two substudies conducting sports and nutritional medical examinations of participants living in the postal code area 79 (South Baden). The protocol of these substudies will be published separately. The IfSS will also evaluate the usage behavior data from the *Increasing Fitness* and *Losing and Maintaining Weight* programs.

## Methods/design

### Study aim, design, and setting

The aim of this study is to test the long-term effectiveness of the *TK-HealthCoach* with regard to these primary outcomes: increased physical activity, weight loss, and smoking abstinence. An online randomized controlled trial (RCT) of parallel design is being conducted as part of the summative evaluation for each of the three health goals *Increasing Fitness*, *Losing and Maintaining Weight*, and *Smoking Cessation*. The aim is to examine if participants of interactive online programs achieve higher effects on study outcomes than participants of non-interactive programs.

Adults across Germany intending to lose weight, increase their fitness or quit smoking are invited to participate in one of the online studies. We are planning to recruit a total of approximately *N* = 2567 participants in the three online RCTs. In addition to these online RCTs, the Department for Sport and Sport Science (IfSS), University of Freiburg is conducting two medical substudies on the health goals *Increasing Fitness* and *Losing and Maintaining Weight*. The aim of these two substudies is to determine long-term physiological effectiveness as a result of behavioral changes. Since the precondition for participating in either substudy is to fill out the online questionnaires, the total number of participants to be recruited has to increase by *n* = 336 to *n* = 2903 (sample size calculation see Table 5).

Formative evaluations will be conducted in addition to the *TK-HealthCoach’*s summative evaluation. These include (a) detailed quantitative analyses of usage behavior data and (b) qualitative interviews with persons participating in the intervention group of the online RCT *Smoking Cessation*.

Study period of each RCT includes the program runtime of the health goals (*Smoking Cessation*: six weeks; *Increasing Fitness* and *Losing and Maintaining Weight*: 12 weeks) as well as another twelve-month follow-up after the program’s completion.

Participant data is being captured via online questionnaires at these time points:

- Baseline t0: after registration and before the program starts
- Follow-up t1: after completing the program
  - *Smoking Cessation*: after 6 weeks
  - *Increasing Fitness* and *Losing and Maintaining Weight*: after 12 weeks
- Follow-up t2:
  - *Smoking Cessation*: 4 months after completing the program
  - *Increasing Fitness* and *Losing and Maintaining Weight*: 6 months after completing the program
- Follow-up t3: 12 months after completing the program in all three RCTs.

The data on program usage will be recorded during the entire study period.

In the RCT *Smoking Cessation* qualitative telephone interviews will be carried out once the t1 questionnaires have been completed.

### Research questions and hypothesis

The following questions and hypotheses are being investigated in this study

1. What effects do the online programs within the three health goals *Losing and Maintaining Weight*, *Increasing Fitness*, and *Smoking Cessation* have on the primary and secondary outcomes variables reported by participants?
  We assume that we will observe statistically significant improvements with small to medium effect sizes for each program.
2. Will the intervention groups (interactive health programs) in each RCT reveal stronger effects on primary and secondary outcomes in comparison to the control groups (non-interactive health programs)? We will test the hypothesis that the intervention groups’ changes in the primary and secondary outcomes are more positive than the control groups’. Our focus is on long-term effects so we will analyze the change between baseline (t0) and 1 year after the intervention finishes (t3), while short-term effects are part of the secondary analyses. We expect small to medium effect sizes from the comparison between these two groups.
3. Does the intensity of use of health programs have an impact on their effects?
  The hypothesis we test is that study participants who use their health program more frequently and intensively achieve better outcomes than study participants who use the program less often or less intensively.
4. How satisfied with the programs offered are the study participants in the intervention- and control groups? This question will be examined exploratively in secondary analyses.

### Study population and recruitment procedure

**Fig. 1:**
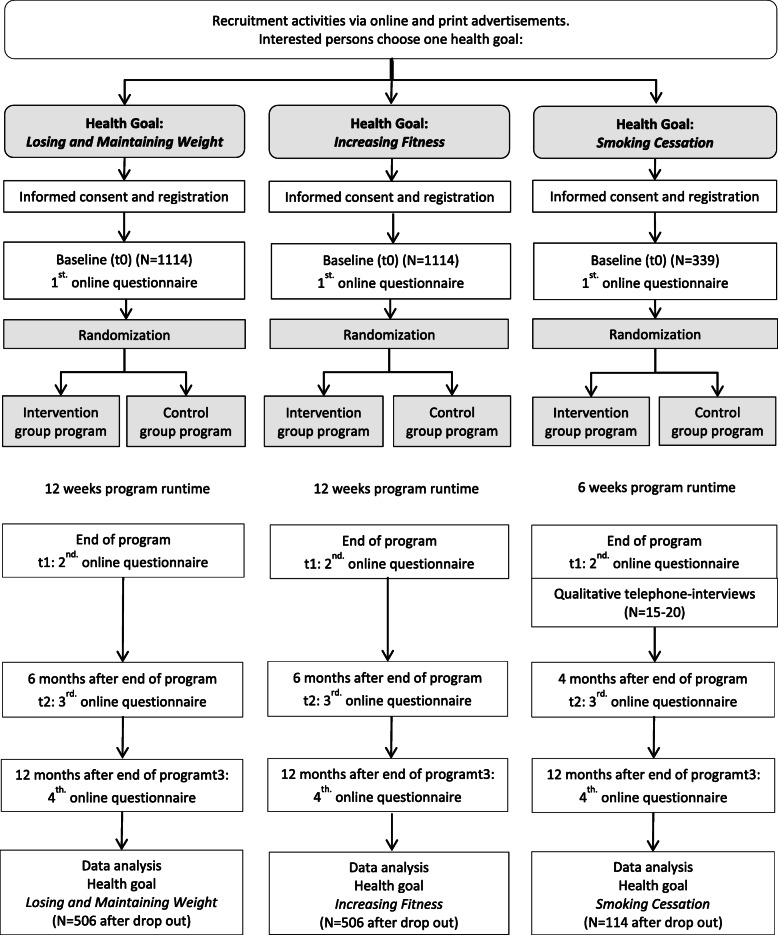
Flowchart of three RCTs to evaluate the *TK-HealthCoach*. See Table 4 for sample size details. *N* = absolute number; *t* = measurement time point

The process of the three online-RCTs, each with parallel groups and equal distribution, is presented in the flowchart (Fig. 1).

### Inclusion criteria

Healthy persons (male/female/diverse*)*, aged 18 years and older, regardless of whether they are insured with *TK health insurance fund*, are eligible to participate. Interested persons with impairments or pre-existing conditions require a medical assessment as to whether the program is suitable for them. Participation is only permitted on the condition that interested persons provide truthful information with regard to their health status. Each person can only choose one health goal and therefore participate in only one RCT.

### Specific inclusion criteria for the “Smoking Cessation” RCT

Only people who smoke at baseline (t0) can take part in the “Smoking Cessation” online RCT.

### Specific inclusion criteria for semi-standardized telephone interviews

Only participants in the “Smoking Cessation” online RCT’s intervention group and those who have completed the t1 online questionnaire can register for these interviews.

### Exclusion criteria

People will be excluded from participating in the program or study if

- Their health impairments which, according to a medical assessment, partially or completely rule out participation in the chosen online health program,
- They are already participating in another study aiming to change behavior towards the health goal in question.

### Specific exclusion criteria for the Losing and Maintaining Weight RCT

- Pregnancy or breastfeeding,
- Waist circumference > 200 cm or body mass index (BMI) > 40 kg/m^2^,
- Currently underweight or underweight after a weight loss of 3 kg,

Interested persons are asked to calculate their BMI (link offered).

### Specific exclusion criteria for the Increasing Fitness RCT

- Pregnancy,
- Currently underweight

Persons with waist circumference > 200 cm or BMI > 40 kg/m^2^ must obtain a medical assessment that allows participation in the health program.

Interested persons are asked to calculate their BMI (link offered).

### Specific exclusion criteria for the Smoking Cessation RCT

Interested persons cannot participate if they have already quit smoking, even if it is only for a few days.

### Specific exclusion criteria for the telephone interview

- Any restrictions making a telephone survey impossible, such as severe communication difficulties.

### Recruitment procedure

From January 1 to 17 of June 2020, a total of *N* = 2903 study participants have to be recruited in all RCTs, including the local medical substudies. The number of participants who have to be enrolled in the online studies is N = 2567.

Recruitment activities and information are being disseminated via online and print media. These include various information provided via channels of the *TK health insurance* (websites, newsletters, blogs etc.), advertisements on Google, and different health media. Advertisements in the local press and radio, the intranet of the University of Freiburg and its Medical Center, and a local display of flyers serve to recruit specifically for the regional medical substudies.

People intending to change their health behavior find brief information about the study and its objectives on the project’s homepage. As specific inclusion and exclusion criteria apply to each of the three programs or RCTs respectively, detailed study information is provided on each program entry.

If interested persons give their informed consent to participate in the program and study, and after completing their registration, they will as participants receive the first health goal-specific questionnaire by e-mail (baseline t0).

Participants in the *Increasing Fitness* or *Losing and Maintaining Weight* RCTs living in the postal code area 79 are offered the possibility to enroll in the medical substudies after filling out the t0 questionnaire. These substudies entail sports and nutritional medical examinations (details will be published in the study protocol of the medical substudies).

### Randomization

After completing the baseline questionnaire, the online RCT participants are given access to their selected online health program. While accessing the program, the participants in each online RCT are allocated at random to the interactive online health program (intervention group) or the non-interactive online health program (control group). Randomizations take place separately for each RCT *Increasing Fitness*, *Losing and Maintaining Weight*, and *Smoking Cessation* via permuting block randomization with variable block sizes of 4, 6, and 8. The participants are assigned to the intervention or control group automatically according to randomization lists. These randomization lists for each RCT were created by SEVERA, applying the software RITA, version 1.50 [19], and transmitted on May 28, 2019, to the IT Company (Vilua Healthcare GmbH), responsible for operating the online program and online survey. The randomization lists cannot be influenced by participants, the IT Company, or the health program’s provider.

We chose permuting block randomization to ensure equal distribution in the intervention and control groups in case of deviations between the numbers of targeted and indeed enrolled participants.

Blinding of the study participants is not possible due to their informed consent to participate in one of two different health-program forms (interactive vs. non-interactive). By participating actively in the interventions, they will be immediately aware of the group to which they have been allocated. Blinded data analysis cannot be ensured either, since the data collected may indicate the study-arm affiliation.

## Interventions

All study participants are given access to a health goal-specific, web-based online program with the difference being that the intervention group is offered a comprehensive, interactive program and the control group a less extensive, non-interactive program.

All participants have access to a personal profile page where they find their personal registration data, study information, and declaration of consent as well as the possibility to unsubscribe from the study and/or program.

### Intervention groups

Participants in the intervention groups are given access to the technically guided, interactive programs *FitnessCoaching*, *WeightLossCoaching*, or *SmokingCessationCoaching*.

The recommended duration of participation in *FitnessCoaching* and *WeightLossCoaching* is 12 weeks, that in *SmokingCessationCoaching* 6 weeks.

The overall aim of each coaching module is to guide users step by step to their health goal and prepare them to continue after the coaching. For this, the users are asked to compile their activities and will then receive a tailored plan and technically guided psychological input to achieve sustainable, positive health effects. Each coaching module contains specific tools to help avoid relapsing. In addition, participants are advised by experts from the IT Company on request.

*Individual action planning*: A common feature of the three coaching modules is their multimodality, interactivity and individual applicability. Each program starts with an anamnesis specific to the chosen health goal. Based on those results, participants plan individual activities with adaptable intensity. All selectable activities are accompanied by video-, audio- or reading instructions. Since many activities benefit different health goals, the participants can choose their individual activities across all three coaching modules—with the exception that the *SmokingCessationCoaching* activities cannot be selected by participants in *FitnessCoaching* or *WeightLossCoaching*.

To enhance health-related knowledge, users are given access to a comprehensive, evidence-based knowledge area divided into different articles. The program pre-filters relevant content for the user. Nevertheless, each user in the intervention group can access all information, regardless of their coaching module.

Weekly planned activities, specific articles, and advice as well as test-tools and daily motivation tips are displayed on an individual dashboard personalized to accommodate each user’s current progress.

*Logging results, feedback, and motivation*: All activities carried out, data from activity trackers, self-tests, nutrition and smoking protocols, as well as read articles, etc., can be logged on by participants. Pop-up toasts or notifications depending on the progress of the participants give feedback on attained goals, motivate user to continue, or suggest specific barrier management. The latter is implemented in the *FitnessCoaching* and the *SmokingCessationCoaching*.

A personal profile page contains an overview of each participant’s progress in weight loss and physical activity or days not smoking. It also contains the study information and informed consent form as well as buttons to withdraw participation in the study or program. At the end of each coaching module (after 6 or 12 weeks, respectively), a review page containing an overview of each participant’s goals and attainments is displayed.

#### Specificities of the coaching modules

- The *WeightLossCoaching* focuses on a sustainable change in diet and the principle of balanced nutrition. The aim is that participants reduce their weight by a maximum of 5 kg within a 12-week period. Its main tool, based on the concept of energy density [20], is a comprehensive protocol of nutrition and physical activity by which an individual’s optimal energy requirements are calculated. An extensive cookbook with recipes is available.
- The *FitnessCoaching* main elements are training plans for strength, endurance, and mobility characterized by progressive training loads through various sports activities such as pilates, back training, yoga, circuit training, Nordic walking, jogging, and cycling. Additional physical activities can be added.
- The *SmokingCessationCoaching* applies the cold-turkey strategy and has a modular structure. Users can get themselves ready for the smoking-cessation date during a preparation phase lasting a maximum 14 days, followed by a 4-week weaning phase. The *SmokingCessationCoaching* includes meditation and mindfulness modules. Additionally, participants are invited to take part in an individual telephone counseling entailing up to four consultations. The counselors are especially trained to carry out this intervention (for details see supplement 1) (Table 1).

**Table 1:** Number of specific components of each coaching module

	Interactive self-tests/courses	Specific activities	Specific articles/videos	Individual tips	Cooking receipts/receipt videos	Physical exercises/videos
			Number *N*			
WeightLossCoaching	3 /1	13	18/18	503	2500/300	
FitnessCoaching	5/1	13	29/20	126		170 /147
SmokingCessationCoaching	4/2		29 /12	32		

The participants can choose their individual activities across all three coaching modules—with the exception that the SmokingCessationCoaching activities cannot be selected by participants in FitnessCoaching or WeightLossCoaching

### Control groups

The control group participants are given access to a non-interactive online health program according to their selected health goal. They receive evidence-based information with advice to attain their goals. This information is divided into different lessons on a website especially designed for each health goal. Study participants are unable to link training plans, protocols or data from activity trackers to the program. A personal profile page contains study information, the informed consent form, and buttons to withdraw participation in the study or program.

After the trial-dependent program ends, all participants, regardless of their study arm, will be given access to the trial-independent interactive *TK-HealthCoach*. This will be possible for all persons regardless of their membership in the *TK health insurance fund*. While the development of each trial-dependent coaching module (used by study participants) was concluded at the beginning of the study, the trial-independent *TK-HealthCoach* has been expanded to incorporate new coaching modules, i.e., *AntistressCoaching*.

### Data collection

Due to summative and formative evaluations, different types of data will be collected in this study. Self-reported outcomes will be surveyed via online questionnaires at all measurement time points (t0, t1, t2, t3). Users withdrawing their participation in the study or the health program prematurely, will receive a short logoff questionnaire upon deregistration to assess reasons for study termination. Usage behavior data will be stored from all participants who log onto their health program, from their first to their last login. Qualitative data will be collected during telephone interviews with participants in the intervention group in the *Smoking Cessation* RCT.

Sports and nutritional medical data will be collected by the IfSS as part of the two substudies. Physiological outcomes will be evaluated in each via four medical examinations (t0, t1, t2, t3). Completing the online questionnaire is a precondition for participating in one of the medical substudies. Detailed information on this data collection will be published in a separate study protocol.

### Quantitative data

#### Online questionnaires: baseline (t0) and catamnesis (t1–t3)

Online questionnaires specifically designed for each health goal are applied in all RCTs to survey socio-demographic, health-related, and psychological variables.

The variables and constructs of the online questionnaire surveys of all RCTs at the four measurement time points are shown in Tables 2, 3, and 4. We used validated instruments whenever possible. The wording in some instruments has been modified and other questions newly developed by the research team. A new questionnaire to investigate eating behavior [21] has been developed as part of the medical preliminary study (details see separate study protocol of the medical substudies [22].

**Table 2:** Variables of three health goal-specific questionnaires: sociodemographic and baseline variables

Variables/constructs	Source/origin/analyses	Health goal /RCT	Number of items	Time measure points			
				t0	t1	t2	t3
**Sociodemography**							
Age	Self-developed/means in years	W, F, S	1	**x**	**x**	**x**	**x**
Gender	Self-developed/percentages	W, F, S	1	**x**	**x**	**x**	**x**
Postal code (2 digits)	Self-developed /percentages	W, F, S	1	**x**	**x**	**x**	**x**
Characteristic of residence (big city to rural)	Self-developed/percentages	W, F, S	1	**x**	**x**	**x**	**x**
Native language	Migration status; adapted [47]/percentages	W, F, S	2	**x**			
Nationality	Self-developed/percentages	W, F, S	1	**x**			
Marital status	Indicators of rehabilitation status (IRES) [48]/percentages	W, F, S	1	**x**			
Persons in the household	IRES [48]/means	W, F, S	2	**x**			
Highest school degree	IRES [48]/percentages	W, F, S	1	**x**			
Highest professional qualification	Questionnaire for Health-Related Resource Use in an Elderly Population FIMA [49]/percentages	W, F, S	1	**x**			
Employment	Indicators of rehabilitation status (IRES); adaped [48]/percentages	W, F, S	2	**x**	**x**	**x**	**x**
Net income	IRES; adaped [48]/percentages of categories	W, F, S	1	**x**			
Working hours	IRE S[48] and one self-developed item/means	W, F, S	2	**x**	**x**	**x**	**x**
**Basic variables**							
Membership in health insurance	Self-developed/percentages	W, F, S	1	**x**			
Knowledge of the study	Self-developed/percentages	W, F, S	1	**x**			
Experience with online health programs	Self-developed/means and percentages	W, F, S	1	**x**			
Current use of (other) online health programs	Self-developed/percentages	W,F,S	6	**x**	**x** (6)	**x**(6)	**x** (6)
Further internet use in general and regarding health subjects	Self-developed/percentages	W, F, S	10	**x**	**x** (1)	**x** (1)	**x**(1)
Previous attempt at behavior change	“Berlin Risk Appraisal and Health Motivation Study” (BRAHMS [50]); adapted to specific health goal/means	W, F, S	2 4	**x**			
Actual health behavior	Self-developed/percentages	W, F, S	1	**x**	**x**	**x**	**x**
Height	IRES [48] (Use for calculation of Body mass index)	W, F, S	1	**x**			
Smoking	Self-developed/percentages	W, F	1	**x**			

Health goals: W=”Losing and Maintaining Weight”; F=”Increasing Fitness”, S=” Smoking Cessation”;

measurement time point: t0 = Baseline, t1 = first follow-up; t2 = second follow-up; t3 = third follow-up; IRES = Indicators of rehabilitation status

**Table 3:** Variables of three health goal-specific questionnaires: primary outcomes and secondary outcomes

Variables/Constructs	Source/Origin/Analyses	Health goal	Number of items	Time measure points			
				t0	t1	t2	t3
**Primary**^**i**^**and secondary**^**ii**^**Outcomes**							
Weight	IRES [48]/Changes in mean kilogram	**W**^**i**^,F^**ii**^,S^ii^	1	**x**	**x**	**x**	**x**
Physical activity	Physical Activity and Sport Activity Questionnaire (BSA-F 3.0) [51]/Changes in mean score	W^ii^,**F**^**i**^	17	**x**	**x**	**x**	**x**
Smoking abstinence in the last 30 days	Self –developed/Changes in percentages (dichotomous data)	**S**^**i**^	1	**x**	**x**	**x**	**x**
Health related goal intention	Goal intention [52] [53]; adapted/Differences of mean values	W^ii^,F^**ii**^,S^ii^	1	**x**	**x**	**x**	**x**
Sport- and movement-related self-concordance	Sport- and movement-related self-concordance [54]/Changes in mean score	F^ii^	12	**x**	**x**	**x**	**x**
Self-efficacy (dietary)	Testing phase-specific self-efficacy [55]/Changes in mean score	W^ii^	7	**x**	**x**	**x**	**x**
Self-efficacy (smoking)	Scales for the measurement of self-efficacy and decisional balance in the process of behavioral change in smokers [56]/Changes in mean score	S^ii^	9	**x**	**x**	**x**	**x**
Barrier management in physical exercise	Barriers and barrier management in physical exercise [57]/Changes in mean score	F^ii^	29	**x**	**x**	**x**	**x**
Expectation of consequences	BRAHMS [50]; adapted/Changes in mean score	W^ii^, S^ii^	18	**x**	**x**	**x**	**x**
Action planning	Action plans and coping plans for physical exercise [58]/Changes in means score	F^ii^	13	**x**	**x**	**x**	**x**
Risk perception	Risk perceptions of cigarettes and e-cigarettes [59]; adapted/Changes in mean score	S^ii^	5	**x**			
Perceived goal attainment	Self-developed/Differences in means	W^ii^,F^ii^,S^ii^	2		**x**	**x**	**x**
Eating behavior German Eating Behavior Scale	German Eating Behavior Scale (SEV)*;article submitted [21]/Changes in mean score	W^ii^, F^ii^	32	**x**	**x**	**x**	**x**
Nicotine dependence	Fagerstrom Tolerance Questionnaire *(*FTND [60])/Changes in mean score	S^ii^	6	**x**	**x**	**x**	**x**
Health-related quality of life	Short Form 12 Health Survey (SF-12) [61]/Changes in mean score	W^ii^,F^ii^,S^ii^	12	**x**	**x**	**x**	**x**

Health goals: W=”Losing and Maintaining Weight”; F=”Increasing Fitness”, S=” Smoking Cessation”

Measurement time point: t0 = baseline, t1 = first follow-up; t2 = second follow-up; t3 = third follow-up; IRES = indicators of rehabilitation status

Primary outcome of the related health goals; highlighted in bold

Secondary outcomes of the related health goals

^*^This instrument (SEV) was developed in the preliminary study [22]

**Table 4:** Variables of three health-specific questionnaires: confounder, use, evaluation, and cancelation of the programs

Variables/constructs	Source/origin/consideration in analyses	Health goal	Number of items	Time measure points			
				t0	t1	t2	t3
**Confounder**							
Mental illness	Patient Health Questionnaire (PHQ-2 [62])/mean score One self-developed item/dichotomous	W, F, S	3	**x**	**x**	**x**	**x**
Health impairments	Comorbidity Score (KoMo) [32]/mean score	W, F, S	24	**x**			
General state of health	BRAHMS [50]; adapted	W, F, S	1	**x**			
Addictive substances other than tobacco (s)	Alcohol, Smoking and Substance Involvement Screening Test (WHO-ASSIST), question N°2 [63] ; three of ten items: alcohol, cannabis, sedatives and sleeping pills/value of each Item	S	3	**x**	**x**	**x**	**x**
Social support	The weigh-related interactions Scale (WRIS) [26]; adapted/mean score	W	14	**x**	**x**	**x**	**x**
	Social support [64] (in prep.)/mean score	F	12	**x**	**x**	**x**	**x**
	Partner Interaction Questionnaire (PIQ-20 [65]); adapted/mean score	S	17	**x**	**x**	**x**	**x**
Evaluation of the program*							
Use and evaluation of the online programs*	Self-developed/mean duration of use in weeks and mean score of evaluation	W, F, S	19		**x**	(x*)	(x*)
Recommendation of the online program	Self-developed/percentages of three levels	W, F, S	1			**x**	**x**
Premature program- and/or study termination**	Self-developed/percentages of different reasons	W, F, S	5	**(x)**			
Text box for additional comments	Self-developed/categorizations and subsequent description of percentages	W, F, S	1	**x**	**x**	**x**	**x**

Health goals: W=”Losing and Maintaining Weight”; F=”Increasing Fitness”, S = “Smoking Cessation”

Measurement time point: t0 = baseline, t1 = first follow-up; t2 = second follow-up; t3 = third follow-up

*These questions are queried only once. If t1- or t2-questionnaire are not filled out by users, these questions are displayed again in the following measurement time point

**Users withdrawing participation in the study or their health program prematurely receive a logoff questionnaire upon deregistration with up to 5 items concerning the reasons for quitting the program and/or study. Additionally, it contains the same questions regarding their usage and evaluation of the program like participants in the t1 online questionnaires. The programming of the questionnaires and databases ensures that participants can only fill in these questions once

Before the online implementation, we pretested the questionnaires for understandability and usability by applying a “think-aloud method” with 11 participants (see supplement 2). Additionally, four of our research team members tested the questionnaires in advance through an expert review. During the process of online implementation and technical realization, two members of the research team monitored the rigorous implementation and web-based display of the questionnaires.

#### Usage behavior data

Usage behavior data will be collected from all participants engaged in the web-based health programs (intervention- and control groups) from their first to their last login. These data are partially collected at the participant level (frequencies and duration of program logins, logged activities [only intervention group]), and partially aggregated (page views).

### Qualitative data

Participants in the *SmokingCessationCoaching* (only participants in the intervention group of the corresponding RCT) will be invited to participate in a telephone interview after completing the t1-questionnaire. The duration of each interview will be 30–45 min.

Program compliance, usage intensity, motivation, and user satisfaction as well as smoking history and sociodemographic data will be surveyed by SEVERA-team researchers posing the following key questions.

*The pretested interview guideline includes the following key questions:*

1. What experiences did you have with the smoking cessation program?
2. How did you feel in the smoking cessation preparation phase?
3. How was your first day not smoking?
4. From your point of view, how was the program’s usability?
5. Did you take advantage of the personal telephone counseling offered in the *SmokingCessationCoaching*? If so, can you tell me about your experiences? If not, why did you not engage with the telephone counseling?
6. What motivated you to begin this non-smoking program (also in comparison to other programs)?
7. How satisfied were you with this non-smoking program and why?
8. Would you recommend this smoking cessation program to others? If so, why would you recommend it, and if not, why would you not recommend it to others?
9. Sociodemographic data: age, gender, native language, graduation, professional qualification, employment, and experience with computer-aided programs.
10. Smoking history
  - Are you currently a smoker?
    i. If no: How long have you not smoked a cigarette?
    ii. If no: How confident are you that you will manage to keep from smoking and why?
    iii. If yes: What are the reasons that you currently smoke?
    iv. If yes: Would you like to go through the program again, or where would you prefer to start?
  - How long did you smoke?
  - How many cigarettes a day did/do you smoke?
  - When did/do you smoke your first cigarette in the morning?
  - Have you tried to quit smoking before? (If so, how often? How successfully?)
  - Had you participated in any other smoking cessation programs before this online program? (If so, which?)
  - Have you used nicotine-replacement products as a cigarette alternative? (If so, which ones?)

### Data management

All quantitative research data are recorded and stored with individual pseudonyms by Vilua Healthcare GmbH (https://vilua.com/services/). Vilua has years of experience managing large datasets related to health programs and research projects and applies high quality standards in terms of data protection and data management.

Since the study started, Vilua sends at regular 2-day intervals datasets to SEVERA which include the number of received baseline-questionnaires in each health goal, randomized users of the programs, as the well as the number of questionnaires of all the following assessment time points distributed, received, and blocked due to exceeding the response time (eight weeks maximum). Due to this monitoring process, the research teams and Vilua are reviewing whether online recruitment and e-mailing of the questionnaires are technically successful. Users are able to contact Vilua the SEVERA and/or IfSS to address technical problems. Possible irregularities are immediately clarified between Vilua and scientific staff members of SEVERA and existing problems (e.g., failures in the storage of usage behavior data, feedback from users about technical errors, etc.) are logged by Vilua, SEVERA, and the IfSS.

*The TK health insurance fund will have no access to this study’s raw data*, *but is only informed about the progress. There exist no competing interests*.

In addition, SEVERA regularly prepares monitoring reports and communicates them to all the collaboration partners. The reports support the decision-making process, shared by the entire project team, to strengthen recruitment efforts and, finally, to complete the recruitment.

Data from the questionnaires are assessed approximately every 14 to 30 days for control purposes. Usage behavior data from the program are transferred at larger intervals. The data transfers are carried out by Vilua via a secure cloud of the Medical Center – University of Freiburg. The data are conducted via comma-separated values (csv) format and merged into the statistic program *Statistical Package for the Social Sciences* (SPSS) data files (IBM SPSS; Version 26), *R* (Version 4.0.3), and *IDE R Studio* (Version 1.2.5033).

After plausibility checks conducted by the scientific staff members of SEVERA, data analyses will be performed by SEVERA and IfSS.

The telephone interviews will be stored digitally as audio files and analyzed by scientific staff of SEVERA. Due to data protection requirements, parts of the interviews including personal information on the participants will be deleted by research assistants. A separate ID will be used to pseudonymize the interviews. The transcriptions of the audio recordings will be conducted by an external office. The data will be transferred via the secured connection to the file server provided by this office. To carry out the qualitative data analysis (QDA), the software MAXQDA (Version2020) will be used.

The *TK health insurance fund* will have no access to this study’s raw data but is only informed about the recruitment progress.

### Study outcomes and data analyses

#### Summative evaluation

Our four major research questions for this project are illustrated above.

We will use a linear mixed model (LMM) for the primary outcomes weight loss (kilogram) and fitness (BSA-Score), and for the outcome smoking cessation, we use a generalized linear model (GLMM) because of the dichotomous outcome variable (Smoking yes/no). The cluster variable is the person-id (level 2-group variable) with the time point nested within it (level 1 measurement), that permits us to model individual and group level changes [23–25]. For each primary outcome, we apply the following basic model with cross-level interaction and random slopes:

Level 1: *Outcome* = *β*_0*j*_ + *β*_1*j*_ ∗ *time* + *β*_*nj*_ ∗ *X*_*nj*_ + *r*_*ij*_

Level 2: *β*_0*j*_ = *γ*_00_ + *γ*_01_ ∗ *group* + *γ*_0*n*_ ∗ *Z*_0*n*_ + *u*_*oj*_

$$ {\beta}_{1j}={\gamma}_{10}+{\gamma}_{11}\ast group+{u}_{1j} $$

“Group” will be a dichotomous variable, which contrasts the intervention group vs. the control group. ’Time’ consists of four time points coded with 0 to 3.

Possible covariates (see Table 4) will enter the model at level 1 (X) when they are measured at more than one time point or at level 2 (Z) when they are only measured at baseline. The covariates differ in type and number between the three coaches. The components X and Z are therefore only placeholders. The covariates considered may represent real-life conditions of participants existing independently of the program; they can be beneficial or detrimental to participants’ goal attainment or outcomes. The social environment in particular may be conducive or an obstacle [26–30]. The importance of social and emotional support from people, who are close, is also pointed out by the NICE guideline *Behaviour Change: individual approaches* [31]. Tiredness and a decrease in drive and activity are indications of mood disorders. Self-efficacy, motivation and intention to change, as well as functional or physical limitations are predictors for behavioral change and associated with mental health, health status, and comorbidities [32–40]. Furthermore, it is likely that additional addictive behaviors and low degree of mental health will make smoking cessation more difficult [33, 41, 42]. Since past behavior predicts future behavior, as confirmed in a meta-analysis [43], preconditions such as mental health, health status comorbidity, additional addictive behavior, but also social support, assessed at baseline, are considered as program-independent covariates of primary outcomes. We use these covariates, along with sex, age, and primary outcome at baseline to adjust the statistical models in the analyses. In these models, coefficient *γ*_10_ refers to the change over time of the intervention groups (hypothesis 1), while coefficient *γ*_11_ refers to the difference in change between intervention and control group (hypothesis 2).

For the analyses of secondary outcomes, measuring mean changes over time (see Table 3), we use linear mixed models analogous to the primary outcomes in weight loss and fitness. Differences between intervention and control groups in for example “Health related goal intention” or “Perceived goal attainment” will be analyzed using *t* tests.

To test the dose-effect association (hypothesis 3), the outlined model will be adapted—here, we only use the intervention group, so the group variable will be dropped. Instead of the group variable, a variable that code the use (e.g., number of logins) of the coach will enter the model at level 2.

All metric covariates at level 1 will be group mean-centered, while metric covariates at level 2 will be grand mean-centered. Categorical variables enter as dummy-coded (0/1) variables.

Post-hoc analyses with correction for multiple testing will be conducted to identify changes between specific time points to analyze short-term effects, e.g., between t0 and t1. As effect size, with their 95% confidence intervals, we will calculate Cohen’s *d* bases on the *t* values from the pairwise comparisons from the LMM respective odds ratio for the GLMM.

In the case of the *SmokingCessationCoaching*, we will drop the baseline (T0) from the model, because every case concerns smoking. It is, therefore, not possible to calculate the odds ratio for the change between baseline and post-intervention. Instead, we will use the difference in smoking cessation at post intervention between both groups as the effect size.

Furthermore, we will apply structural equation modeling to test various models for assessing potential mediators of the intervention effect on outcomes.

The analyses are carried out according to the intention-to-treat principle (ITT). In ITT, all randomized participants will be considered in the main analyses. In per-protocol analyses (PP), only those participants will be included who have been randomized, have participated at all time points, and filled out corresponding primary outcomes. The randomization condition for study participants was that they accessed the program at least once.

#### Sensitivity analysis and missing values

We will compare the results from intention-to-treat (ITT) analyses with per-protocol analyses (PP) and drop-out analyses. We test if participants, who have continuously taken part in the online survey, achieve better outcomes. Due to different procedures to impute missing values (see below), we will use linear mixed models, (a) with imputed covariates on ITT and PP analyses and (b) without covariates in ITT and PP analyses to compare the raw change in outcomes over time.

In addition, we will consider data on program use, (a) between intervention- and control-groups and (b) within intervention groups, between participants who use the program only once and those who use it multiple times.

In the missing value analysis, we analyze the patterns of missing values as well as frequency for cases and variables. The missing values will be imputed using multiple imputation (MI) technique, if the assumption of missing at random (MAR) or missing complete at random (MCAR) holds.

The imputation approach differs between ITT and PP because in PP, we only include participants without missing values on outcome criteria in the analysis. Therefore, only missing values on predictor variables will be imputed, but not for the outcome variables, while in the ITT analysis, predictor variables as outcome variables will be imputed too.

#### Formative evaluation

Our fourth and final investigative aim is to characterize the intervention- and control-group participants’ ways of use and satisfaction with their corresponding online program as further secondary outcomes. To that end, the first step will be to conduct a descriptive analysis; in step 2, we will test via regression models our hypotheses regarding predictors of satisfaction.

The telephone interviews with users of the *SmokingCessationCoach* (intervention group of the corresponding RCT) will be analyzed taking a qualitative content analysis approach. The transcripts of the interviews will be coded and analyzed according to a deductive and inductive approach. We will form main- and subcategories out of the identified themes. Deductive main categories will correspond to the main topics of the interview guideline. Inductive categories will result from additional content mentioned by the participants in the interviews. Subcategories will form subordinate topics or different ratings of a main category. According to the categorization system, transcripts will be coded, ordered, summarized and systematically interpreted [44]. For illustration purposes, the presentation of the results will include individual quotes of the participants.

### Sample size calculation

#### Randomized controlled trials

We calculated the sample size for each randomized controlled trial (RCT) separately (see Table 5) by considering the effect sizes of the primary outcomes in comparable trials [8–11], the α-error (0.05), and the power we were striving for (1 − *β* = 0.80). Our power analyses were done using GPOWER software. While we assumed a general online-RCT dropout rate of 50% [45, 46], we estimate a dropout rate of about 15% in the medical substudies in light of experiences with similar studies involving personal medical examinations. We have accounted for an additional 10% buffer for the online RCTs.

**Table 5:** Sample size calculations for the three RCTs including medical substudies

Health goals	“Increasing Fitness“		“Losing and Maintaining Weight”		“Smoking Cessation“
RCTs	Online questionnaire study	Online-questionnaire study + medical substudy:	Online questionnaire study	Online-questionnaire study + medical substudy:	Online questionnaire study
**Primary outcome**	Physical activity (BSA)	questionnaire study: physical activity (BSA); medical examinations: VO_2 max_	Weight loss	Questionnaire study + medical examinations: weight loss	Abstinence from smoking
**Effect assumption for primary outcome**	effect size = 0.25	questionnaire study: effect size = 0.25 ; medical examinations: effect size = 0.45	Effect size = 0.50	Effect size = 0.50	15% in intervention group versus 2% in control group
**Required case-number results** (two-sided, alpha = 0.05, beta = 0.80)	253 (for each intervention group IG and control group CG)^1^	79 (for each IG and CG)^2^	253 (for each IG and CG)^1,3^	64 (for each IG and CG)^2^	77 (for each IG and CG)
**(assumed dropout)** **minimally required sample size**	(50%) 506 (for each IG and CG)	(15%) 93 (for each IG and CG)	(50%) 506 (for each IG and CG)	(15%) 75 (for each IG and CG)	(50%) 154 (for each IG and CG)
**total sample size (IG + CG + ca. 10% buffer for questionnaire study**	(506 + 506) × 1.1 *N* = 1.114	(93 + 93) *N* = 186	(506 + 506) × 1.1 *N* = 1.114	(75 + 75) *N* = 150	(154 + 154) × 1.1 *N* = 339

IG = intervention group (specific online coach for the health goal chosen), CG = control group (specific health information for the health goal chosen)

^1^As we aimed to assess the groups undergoing additional medical exams separately from those only completing the online questionnaire, the former group’s sample size is not included in this calculation

^2^As the questionnaire study’s outcomes are secondary for this group, our required sample size refers to the medical exam’s outcome

^3^As physical activity is a key secondary outcome for the Lose Weight group, we have considered both primary and secondary outcomes (physical activity) in determining the required sample size

#### Interviews

We plan to conduct 15 to 20 interviews with participants in the RCT *Smoking Cessation* intervention group. This calculation is based in particular on previous experience and is expected to lead to saturation of information. We further assume that this number of users will agree to participate in a telephone interview (approx. 20% of the users in the RCT *Smoking Cessation* intervention group). If further information is required after 15 interviews, up to 5 more interviews will be conducted.

#### Incentives

After the trial-dependent program ends, all participants, irrespective of their study arm, will be given access to the trial-independent, interactive *TK-HealthCoach*. This is possible for all study participants regardless of membership in the *TK health insurance fund*. While the development of the trial-dependent programs (those used for study participants) was stopped at the beginning of the study, the trial-independent *TK-HealthCoach* is being expanded with new Coaching modules such as *AntistressCoaching*.

Participants who filled out all four questionnaires will receive a shopping voucher worth € 25. For returning the t1 and t2 questionnaires participants will receive discount coupons for the purchase of activity trackers. Interview partners will receive in addition a shopping voucher worth € 30. All vouchers will be sent by e-mail.

## Discussion

Main objective of this study is to analyze the effectiveness of the *LosingWeightCoach*, *FitnessCoach*, and *SmokingCessationCoach* health programs by comparing their respective intervention and control groups. We assume that study participants can improve their health behavior by engaging in the offered health programs and that each health program’s intervention group will reveal a benefit in attaining primary and secondary outcomes in comparison to control groups.

The results of our formative evaluation including the responses in the logoff questionnaires, drop-out analysis, broad usage behavior data, and qualitative interviews will enrich our interpretation of the summative results and enable detailed insight into the satisfaction and usability of the programs. Finally, this study will be essential to developing quality criteria for web-based health programs.

### Trial status

Protocol version number 3; date of approval: 2019 July 25 (amendment: 2019 October 28).

Study enrollment began on January 1, 2020. After the first 6 weeks, we noticed that the *Losing and Maintaining Weight* health goal was chosen much more often than the *Increasing Fitness* health goal. As we had expected, recruitment for the *Smoking Cessation* health goal was continuous and good. We therefore tried to draw more attention to the *Increasing Fitness* health goal by adjusting keywords and budget of the online ads.

In line with our study’s risk management, we consider that, as studies involving digital interventions and digital surveys are notorious for encouraging a rather low level of personal commitment, the risk of program drop-outs or study discontinuations is higher than in trials involving personal, face-to-face contacts [45, 46]. In addition, common technical problems on the part of the provider or user can increase dropout rates. For these reasons, individuals interested in the study and its participants are able to contact the participating institutions by phone and/or e-mail to obtain personal advice. Nevertheless, the dropout rate could exceed the estimated 50% rate associated with all online RCTs. Dropouts could occur if participants are dissatisfied with the programs or in case of unpredictable events such as the rapid spread of the coronavirus in Germany since March 2020 and potential problems associated with general high attention, the health of participants, technical problems due to the congestion in the internet, etc. Therefore, we are monitoring the response rates at t1 and, if necessary, will raise the number of participants to be recruited. We closed recruitment on 05 October 2020.

## Supplementary Information

**Additional file 1:.**

**Additional file 2:.**

**Additional file 3:.**

**Additional file 4:.**

**Additional file 5:.**

**Additional file 6:.**

**Additional file 7:.**

## Data Availability

The datasets generated and analyzed during the current study will neither be shared with scientists outside this cooperation project nor with other third parties. The reason for this decision is that it cannot currently be guaranteed that anonymized data will not be de-anonymized in the future because of various data available on the internet. Therefore, the collected data will remain under control of the cooperation partners in this project. Data will be anonymized three years after completion of the study and the research data will be deleted after 10 years. PL declares that he receives a fee from the *TK* for technical support for the consultant interface and for evaluating the *SmokingCessationCoaching*; this includes travel expenses. PL has no connections to the tobacco, e-cigarette, or pharmaceutical industry. There are no other conflicts of interest. RB declares that he receives a fee from TK for cooperating on health issues in the TK’s department of Health Care Management. In this context, RB’s travel expenses are reimbursed. There are no other conflicts of interest.

## Abbreviations

BMI: Body mass index
BRAHMS: Berlin Risk Appraisal and Health Motivation Study
BSA: Physical Activity and Sport Activity Questionnaire
CG: Control group
csv: Comma-separated values; file format
DFG: German Research Foundation
DRKS: German Clinical Trial Register
€: Euro
F: *Increasing Fitness*
SEV: German Eating Behavior Scale
FIMA: Questionnaire for Health-Related Resource Use in an Elderly Population
FTND: Fagerstrom Tolerance Questionnaire
GDPR: European General Data Protection Regulation
GPOWER: Software used to carry out the sample size calculation
ID: Identification number
IfSS: Department of Sport and Sport Science
IG: Intervention group
IRES: Indicators of Rehab Status Questionnaire
JCA: Joint Controller Agreement
KoMo: Comorbidity Score
M: Mean
Max: Maximum
MAXQDA: Software used to carry out the qualitative data analysis
Min: Minimum
N/n: Absolute number
PHQ-2: Patient Health Questionnaire
PIQ-20: Partner Interaction Questionnaire
QDA: Qualitative data analysis
RCT: Randomized controlled trial
RITA: Randomization In Treatment Arms; randomization program for clinical trials used to carry out the randomization lists
S: Smoking cessation
SD: Standard deviation
SEVERA: Section of Health Care Research and Rehabilitation Research
SF-12: Short Form 12
SPSS: Statistical Package for the Social Sciences, software used to carry out statistical analyses
t0, t1, t2, t3: Measurement time point 0 (baseline) and follow ups 1, 2, and 3
TK: *Techniker health insurance fund*
UTN: Universal trial number
VO_2_: Oxygen saturation
vs: Versus
W: Losing and Maintaining Weight
WHO-ASSIST: Alcohol, Smoking and Substance Involvement Screening Test

## Declarations

### Harms and risk

Participation in the online studies and the online health programs are not expected to cause harms or risks—especially considering the inclusion and exclusion criteria with which the users have to agree. If participants have any questions or feel uncomfortable, they can always contact the program's customer service or the study contact persons. All inquiries and possible health problems will be logged. In case of health problems, the principal medical doctor of the study (Prof. Dr. med. D. König) will be informed. The procedure for dealing with very unlikely but possible risks during the medical examinations in the two substudies has been approved by the Ethics Committee Freiburg and will be published elsewhere.

### Publication rules

The main results of studies concerning the effectiveness of health programs are to be published in scientific journals. These publications will be prepared under the leadership of SEVERA, which, as the main institution responsible for evaluation, will also be responsible for data analysis. The other scientific project participants will be involved in these publications. All scientific project participants aim to publish further publications on questions of interest, for which appropriate authorships will be determined according to the usual rules.
