# Supplementary material for: Effectiveness of an interactive web-based health program for adults: a study protocol for three concurrent controlled-randomized trials (EVA-TK-Coach)
Source: Trials. 2021 Aug 10;22:526. doi: 10.1186/s13063-021-05470-8 (PMC8353439; doi:10.1186/s13063-021-05470-8)
Supplement: Supplementary file 1 — Additional file 1:. [file 13063_2021_5470_MOESM1_ESM.docx]

**Supplement: telephone counselling within the „SmokingCessationCoaching“**

Three persons (2 female, 1 male) with background of a medical education conduct the telephone counselling. All have completed a special training for the counselling with a total of 48 hours that included:

- 12 hours: basics on telephone advice on smoking cessation
- 20 hours: curriculum "tobacco dependence & smoking cessation", certified by the scientific working group in smoking cessation (WAT) e.V.
  - 12 hours: specific training related to the “SmokingCessationCoaching”
  - 4 hours: simulation talks
