# Supplementary material for: Effectiveness of an interactive web-based health program for adults: a study protocol for three concurrent controlled-randomized trials (EVA-TK-Coach)
Source: Trials. 2021 Aug 10;22:526. doi: 10.1186/s13063-021-05470-8 (PMC8353439; doi:10.1186/s13063-021-05470-8)
Supplement: Supplementary file 2 — Additional file 2:. [file 13063_2021_5470_MOESM2_ESM.docx]

**Description: Pretest of the online questionnaires with the “think aloud” method**

| Number of tested questionnaires | 11 (Desktop: 7, Mobile 4) |
| --- | --- |
| Losing and Maintaining Weight | 3 (Desktop: 1, Mobile: 2) |
| Increasing Fitness | 5 (Desktop: 4, Mobile: 1) |
| Smoking Cessation | 3 (Desktop: 2, Mobile: 1) |
| Characteristics of participants |  |
| Number of participants | 11 |
| Age | M=31.00; SD=14.97; Min.=18, Max.=68 |
| Gender | Female: 6 (54.5%); Male: 5 (45.5%) |
| Education | University entry level 11 (100%) |
| Current profession | Research associate 4 (36.4%); student 3 (27.3%); pensioner 1 (9.1%); travel agent 1 (9.1%); engineer 1 (9.1%); unclear 1 (9.1%) |

Notes: Desktop and Mobile describe display formats;
M= mean; SD= standard deviation; Min.= minimum; Max.= maximum.

Each participant tested one questionnaire with a “think aloud method”. Additionally 4 people from the research team (IT, GM, CS und RW) tested the questionnaires.
