# Supplementary material for: Effectiveness of an interactive web-based health program for adults: a study protocol for three concurrent controlled-randomized trials (EVA-TK-Coach)
Source: Trials. 2021 Aug 10;22:526. doi: 10.1186/s13063-021-05470-8 (PMC8353439; doi:10.1186/s13063-021-05470-8)
Supplement: Supplementary file 3 — Additional file 3:. [file 13063_2021_5470_MOESM3_ESM.docx]

| **Variables / Constructs (HG)** | **Source / Origin** | **Questions** | **Answer options** |  |
| --- | --- | --- | --- | --- |
| *Sociodemography* | | | | |
| Age (W,F,S) | Self-developed | How old are you (in years)? | [double-digit number field] |  |
| Gender (W,F,S) | Self-developed | Your Gender | female  male  divers | 1 2 3 |
| Postal code (W,F,S) | Self-developed | Please enter the first two numbers of your postal code. | [double-digit number field] |  |
| Characteristic of residence (W,F,S) | Self-developed | How would you describe the characteristics of your place of residence? | rural  small-town  medium town  urban | 1 2 3 4 |
| Native language (W,F,S) | Migration status; adapted [23] | (1) Is German your mother tongue? | Yes  No | 1 2 |
|  |  | (2) If German is not your mother tongue – how do you estimate your German language skills? | not at all  little  average  good  very good | 1 2 3 4 5 |
| Nationality (W,F,S) | Self-developed | What is your nationality?  *(multiple answers possible)* | German  Austrian  Swiss German  Other [50 characters] | 1 2 3 4 |
| Marital status (W,F,S) | Indications of rehabilitation status (IRES) [24] | What is your marital status? | single  in a relationship  married  divorced/separated  widowed | 1 2 3 4 5 |
| Persons in the household (W,F,S) | IRES [24] | How many people including yourself live in your household? Please count your children as well. | In total [two-digit numerical format] person(s)  Thereof XX [two-digit numerical format] person(s) under the age of 18 |  |
| Highest school degree (W,F,S) | IRES [24] | What is your highest school degree? | Primary School or Secondary School Certificate  Middle School Qualification/Secondary School Certificate  Polytechnic Secondary School  Polytechnic degree (Upper Secondary Education)  A-levels / Higher education entrance diploma  Other school leaving qualification  No school leaving qualification | 1 2  3 4  5 6 7 |
| Highest professional qualification (W,F,S) | Questionnaire for Health-Related Resource Use in an Elderly Population FIMA [25] | What is your highest professional qualification? | Vocational School (Apprenticeship)  Vocational School / Technical School / School for craftsmen  Engineering School / Polytechnic  Higher Education Institution /  University of Applied Sciences / University  Other qualification  No qualification | 1  2 3 4  5 6 |
| Employment (W,F,S) | IRES [24] | Are you employed? (Anyone who is currently unable to work or on sick leave is also considered to be employed) | Yes, I am employed  No, I am not employed but  unemployed  vocational training/retraining  Housewife/househusband  Early retirement  Limited employment or occupational disability pension  Unlimited employment or occupational disability pension  Retirement pension  Partial pension  Other | 1   2 3 4 5 6  7  8 9 10 |
| Net income (W,F,S) | IRES [24] | What is the total monthly net income of your household? This information is very useful for statistical purposes, but not essential. | up to 500 Euro  500 up to 1,000 Euro  1,000 up to 2,000 Euro  2,000 up to 3,000 Euro  3,000 up to 4,000 Euro  4,000 up to 5,000 Euro  5,000 Euro and more  I don‘t know | 1 2 3 4 5 6 7 8 |
| Working hours (W,F,S) | IRES [24] | (1) How many hours do you currently work in your profession? (Please also include regularly occurring extra hours) | [three-digit number format] hours per week |  |
|  | Self-developed | (2) Are you working in shifts? | Yes  No | 1 2 |
| *Basic variables* | | | | |
| Membership in health insurance (W,F,S) | Self-developed | Which health insurance company are you a member of? (Please refer to your main insurance company) | TK  BARMER  DAK  AOK  IKK  Knappschaft  KKH  BKK  LKK  Private Health Insurance  Other [50 digits] | 1 2 3 4 5 6 7 8 9 10 11 |
| Knowledge of the study (W,F,S) | Self-developed | How did you hear about this health study?  (*multiple answers are possible*) | Website of TK  TK-newsletter  Customer Service of TK  Facebook  Twitter  Urban or regional website / portals  Local (for free) newspaper  Apotheken Umschau (free health magazine)  (Lifestyle-) magazine  postcards / flyer  General Practitioner  Sportsclub  Other [50 digits] | 1 2 3 4 5 6 7 8 9 10 11 12 13 |
| Experiences with online health programs (W,F,S) | Self-developed | Have you previously participated in an **online**-health program?  (*multiple answers are possible*) | No  Yes, an offer of TK  Yes, an offer of another provider | 1 2 3 |
| Current use of (other) online health programs | Self-developed t | Are you **currently** participating in an **online**-health program? (*multiple answers are possible*)  If so |  |  |
|  |  | (1) On the subject of losing weight | Yes  No | 1 2 |
|  |  | If “yes“: Is that an offer from TK? | Yes  No | 1 2 |
|  |  | (2) On the subject of stress | Yes  No | 1 2 |
|  |  | If “yes“: Is that an offer from TK? | Yes  No | 1 2 |
|  |  | (3) On the subject of fitness | Yes  No | 1 2 |
|  |  | If “yes“: Is that an offer of TK? | Yes  No | 1 2 |
|  |  | (4) On the subject of burnout | Yes  No | 1 2 |
|  |  | If “yes“: Is that an offer of TK? | Yes  No | 1 2 |
|  |  | (5) On the subject to stop smoking | Yes  No | 1 2 |
|  |  | If “yes“: Is that an offer of TK? | Yes  No | 1 2 |
|  |  | (6) On another subject | Yes  No | 1 2 |
|  |  | If “yes“: Is that an offer of TK | Yes  No | 1 2 |
| Further internet use in general and regarding health subjects  Further internet use in general and regarding health subjects | Self-developed  Self-developed | (1) Which further information or offers do you use for your health?  (*multiple answers are possible*) | None at all  Information from the doctor  Information from the pharmacist  Information from the health insurance  Digital media (e.g. web search engines on the internet)  Health information in brochures, guides or similar  TV (e.g. documentaries)  Expert literature  Self-help groups  Courses (e.g. adult education center)  Structured programs (e.g. Weight Watchers)  Activity trackers (e.g. smartwatch, pedometers)  Other (200 characters) | 1  2  3  4  5  6  7  8  9  10  11  12  13 |
|  |  | (2) How often do you use the internet? | Rarely / Never  Several times a month  Several times a week  Daily  Several times a day | 1 2 3 4 5 |
|  |  | (3) How do you use the internet? (“professionally“ includes the usage for school, study, work-related further training) | mainly private  mainly on business  on business as well as private | 1 2 3 |
|  |  | (4) I use digital media (e.g. Internet, apps) to search for health information | strongly disagree  disagree  somewhat disagree  somewhat agree  agree  strongly agree | 1 2 3 4 5 6 |
|  |  | I search health information via the following digital offers: |  |  |
|  |  | (5) Web search engines (e.g. Google) | never  rarely  sometimes  often  very often | 1 2 3 4 5 |
|  |  | (6) Health portals (e.g. Onmeda) |  |  |
|  |  | (7) Social networks as YouTube or Facebook, etc. |  |  |
|  |  | (8) Apps |  |  |
|  |  | (9) Other [50 characters] |  |  |
|  |  | (10) I know how to get the health information that I need. | strongly disagree  disagree  somewhat disagree  somewhat agree  agree  strongly agree | 1 2 3 4 5 6 |
| Previous attempt at behavior change (W) | “Berlin Risk Appraisal and Health Motivation Study” (BRAHMS [26]); adapted to specific health goal | (1) Have you ever tried to change your diet to lose weight? | No  Yes, 1 time  Yes, 2 times  Yes, 3 times  Yes, 4 times  Yes, 5 times or more often | 1 2 3 4 5 6 |
|  |  | (2) If “yes“: When was the last attempt? | [two-digit number format] years ago  [two-digit number format] months ago  I am currently doing it | 1 2 3 |
| Previous attempt at behavior change (F) | BRAHMS [26]; adapted to specific health goal | (1) Have you ever tried to get active again? | No  Yes, 1 time  Yes, 2 times  Yes, 3 times  Yes, 4 times  Yes, 5 times or more often | 1 2 3 4 5 6 |
|  |  | (2) If „yes“: When was the last attempt? | [two-digit number format] years ago  [two-digit number format] months ago  I am currently doing it | 1 2 3 |
| Previous attempt at behavior change (S)  Previous attempt at behavior change (S) | BRAHMS [26]; adapted to specific health goal  BRAHMS [26]; adapted to specific health goal | (1) How often have you seriously tried to stop smoking (at least for 24 hours)? | Not yet  1 time  2 times  3 times  4 times  5 times or more | 1 2 3 4 5 6 |
|  |  | (2) How long was your longest smoke-free period?  *(Please enter the number in the field and click the button of the corresponding time unit)* | [three digit number format]  years  months  weeks  days | 1 2 3 4 |
|  |  | (3) When was your last serious attempt to stop smoking for 24 hours? *(Please enter the number in the field and click the button of the corresponding time unit)* | [three digit number format]  years ago  months ago  weeks ago  days ago | 1 2 3 4 |
|  |  | (4) Have you used tools or did you get support during previous attempts to stop smoking? (multiple answers are possible) | Yes, support from partner / family  Yes, medical consultation  Yes, weaning cure  Yes, nicotine-based products (e.g. nicotine plasters, chewing gums, spray)  Yes, electric cigarette  Yes, tobacco heater (e.g. shisha)  Yes, prescription medication (e.g. Champix®, Zyban®)  Yes, offers as hypnosis or acupuncture  Yes, guidebooks, brochures, etc.  Yes, online-offers  Yes, telephone consultation  Yes, other aids  No | 1 2 3 4  5 6 7  8 9 10 11 12 13 |
| Actual health behavior (W) | Self-developed | Have you regularly paid attention to a calorie-conscious or healthy diet in the last 4 weeks? | No  Yes, with disruption  Yes, without disruption | 1 2 3 |
| Actual health behavior (F) |  | Have you been regularly active in sports during the last 4 weeks? | No  Yes, with disruption  Yes, without disruption | 1 2 3 |
| Actual health behavior (S) |  | Have you smoked **regularly** in the last three months? | No  Yes, with disruption  Yes, without disruption | 1 2 3 |
| Height (W,F,S) | IRES [24] | How tall are you? | [three digit number field] centimeter |  |
| Smoking (W,F) | Self-developed | Do you smoke? | No, never  Yes, sometimes  Yes, regularly | 1 2 3 |
| HG = health goals; Health goals: W=*Losing and Maintaining Weight*; F=*Increasing Fitness*, S=*Smoking Cessation* | | | | |
