# Supplementary material for: Effectiveness of an interactive web-based health program for adults: a study protocol for three concurrent controlled-randomized trials (EVA-TK-Coach)
Source: Trials. 2021 Aug 10;22:526. doi: 10.1186/s13063-021-05470-8 (PMC8353439; doi:10.1186/s13063-021-05470-8)
Supplement: Supplementary file 4 — Additional file 4:. [file 13063_2021_5470_MOESM4_ESM.docx]

| **Variables / Constructs HG** | **Source / Origin** | **Questions** | **Answer options** |  |
| --- | --- | --- | --- | --- |
| *Primary and Secondary Outcomes* | | | | |
| Weight (W,F,S) | Indications of rehabilitation status (IRES) [24] | How much do you weigh without clothes? | [three digit number field] kilogram |  |
| Smoking abstinence in the last 30 days (S) | Own development | Have you smoked tobacco (e.g. cigarettes, cigars, cigarillos) during the past 30 days, even if it was only a puff? | Yes  No | 1 2 |
| Health goal intention (W) | Goal intention [28] [29] - adapted | How strong is your intention to eat a calorie-conscious or rather a healthy diet in the next weeks or months? | Scale 0-5  0 = "I have **no** such intention“  5 = “I have this intention **very strongly**“ |  |
| Health goal intention (F) | Goal intention [28] [29] – adapted | How strong is your intention to get active in sports in the coming weeks and months? | Scale 0-5  0 = “I have **no** such intention“  5 = “I have this intention **very strongly**“ |  |
| Health goal intention (S) | Goal intention [28] [29] - adapted | How strong is your intention to stop smoking? | Scale 0-5  0 = “I have **no** such intention“  5 = “I have this intention **very strongly**“ |  |
| Sport- and movement-related self-concordance (F) | Sport- and movement-related self-concordance [30] | I have the intention to be regularly active in sports during the next weeks or months... |  |  |
|  |  | (1) ...as I enjoy doing sports | disagree  somewhat agree  somewhat disagree  agree | 1 2 3 4 |
|  |  | (2) ...as it is good for me. |  |  |
|  |  | (3) ...as otherwise I would feel guilty. |  |  |
|  |  | (4) …as the positive effects are simply worth the effort |  |  |
|  |  | (5) ...as people who are important to me are pushing me to do sports. |  |  |
|  |  | (6) ...as I gain experiences that I would not like to miss. |  |  |
|  |  | (7) ...as I think that sometimes you have to force yourself to do something. |  |  |
|  |  | (8) …as physical exercises are simply part of my life. |  |  |
|  |  | (9) ...as I otherwise would have to blame myself. |  |  |
|  |  | (10) ...as other people tell me to be active in sports. |  |  |
|  |  | (11) ...as I have good reasons for doing so. |  |  |
|  |  | (12) ...as otherwise I get into trouble with other people. |  |  |
| Self-efficacy (S) | Scales for the measurement of self-efficacy and decisional balance in the process of behavioral change in smokers [32] | I am optimistic not to smoke even when.... |  |  |
|  |  | (1) ...I am with friends at a party. | Scale 1-5 1 = not at all confident 5 = extremely confident |  |
|  |  | (2) ...I am about to get up in the morning. |  |  |
|  |  | (3) ...I feel quite anxious and stressed out. |  |  |
|  |  | (4) ...I drink coffee or tea whilst relaxing. |  |  |
|  |  | (5) ...I feel that I need a cheering up. |  |  |
|  |  | (6) ....I am quite upset about something or someone. |  |  |
|  |  | (7) ...I am with my partner or a good friend and he or she is smoking. |  |  |
|  |  | (8) ...I realize that I haven’t smoked for a while. |  |  |
|  |  | (9) ...things don’t work out the way I want. |  |  |
| Barrier management in physical exercise (F)  Barrier management in physical exercise (F) (Continuation)  Barrier management in physical exercise (F) (Continuation) | Barriers and barrier management in physical exercise [33]  Barriers and barrier management in physical exercise [33]  Barriers and barrier management in physical exercise [33] | How often do the following barriers prevent you from doing sports? |  |  |
|  |  | (1) The weather is bad. | (almost) never sometimes often (almost) always  (almost) never sometimes often (almost) always  (almost) never sometimes often (almost) always | 1 2 3 4  1 2 3 4  1 2 3 4 |
|  |  | (2) I am tired. |  |  |
|  |  | (3) Friends want to meet up with me. |  |  |
|  |  | (4) I am sick. |  |  |
|  |  | (5) I don’t feel like it. |  |  |
|  |  | (6) There is still a lot of work to be done. |  |  |
|  |  | (7) I am in pain. |  |  |
|  |  | (8) It is cosy at home. |  |  |
|  |  | (9) I am in a bad mood. |  |  |
|  |  | (10) I am hurt. |  |  |
|  |  | (11) A good film or show is on television. |  |  |
|  |  | (12) I feel depressed. |  |  |
|  |  | (13) My partner does not want me to do sports. |  |  |
|  |  | (14) I feel stressed out. |  |  |
|  |  | Counter strategies: What are you doing to overcome the just mentioned barriers?  In order keep the sport appointment… |  |  |
|  |  | (15) ...I make an appointment with a friend for regular sports activities. |  |  |
|  |  | (16) ...I put my sports gear within easy reach. |  |  |
|  |  | (17) ...I write down my appointments (e.g. in my calendar). |  |  |
|  |  | (18) ...I intend to treat myself with something nice afterwards. |  |  |
|  |  | (19) ...I avoid situations that prevent me from doing sports (e.g. not to turn on the television). |  |  |
|  |  | (20) I consider my sport appointment as important as other appointments. |  |  |
|  |  | (21) ...I take part in sport activities at an association or gym. |  |  |
|  |  | (22) ...I remind myself of the advantages of doing sports. |  |  |
|  |  | (23) ...I try to put myself in a mood in which I feel like physical activity. |  |  |
|  |  | (24) ...I buy sportswear in which I feel comfortable. |  |  |
|  |  | (25) I don’t even start to think about what I could do instead of sport |  |  |
|  |  | (26) ...I tell my friends/acquaintances about my sport projects. |  |  |
|  |  | (27) ...I look for a sports program that is easy to reach. |  |  |
|  |  | (28) ...I think at my guilty conscience that I’d have if I didn’t go to sports. |  |  |
|  |  | (29) I try to see uncomfortable situations (e.g. fear of disgrace, bad weather) as a challenge. |  |  |
| Expectation of consequences (W)  Expectation of consequences (W) (Continuation) | “Berlin Risk Appraisal and Health Motivation Study” (BRAHMS [26]); adapted to specific health goal  BRAHMS [26] – adapted to specific health goals | If I eat a calorie-conscious or healthy diet (or would eat calorie-conscious or healthily)… |  |  |
|  |  | (1) …that’s good for my health. | disagree  somewhat disagree  somewhat agree  agree  disagree  somewhat disagree  somewhat agree  agree | 1 2 3 4  1 2 3 4 |
|  |  | (2) ... then my family has to take me into consideration. |  |  |
|  |  | (3) ...I feel physically more attractive. |  |  |
|  |  | (4) ...I have no weight issues (anymore). |  |  |
|  |  | (5) ...the food does not taste as good anymore. |  |  |
|  |  | (6) ...my social life is affected (at parties, at an association, with friends). |  |  |
|  |  | (7) ...I prevent a heart attack. |  |  |
|  |  | (8) ...that’s good for my blood pressure. |  |  |
|  |  | (9) ...I feel more at ease. |  |  |
|  |  | (10) ...I need to make an effort to buy the right products. |  |  |
|  |  | (11) ...I feel strongly restricted when I eat. |  |  |
|  |  | (12) ...that’s good for my blood values (cholesterol) |  |  |
|  |  | (13) ...I am a good role model (e.g. for my partner or my children). |  |  |
|  |  | (14) ...then I have to take more time to prepare food. |  |  |
|  |  | (15) ...I am less likely to get sick. |  |  |
|  |  | (16) ...that means a loss of quality of life for me. |  |  |
|  |  | (17) ...is a financial burden for me. |  |  |
|  |  | (18) …other people value my willpower. |  |  |
| Expectation of consequences (S)  Expectation of consequences (S) (Continuation) | BRAHMS [26] – adapted to specific health goal  BRAHMS [26] – adapted to specific health goal | If I don’t smoke (or would stop smoking)… |  |  |
|  |  | (1) ...I am nervous and unbalanced. | disagree  somewhat disagree  somewhat agree  agree  disagree  somewhat disagree  somewhat agree  agree | 1 2 3 4  1 2 3 4 |
|  |  | (2) ...that has a positive effect on my blood pressure. |  |  |
|  |  | (3) ...I save a lot of money. |  |  |
|  |  | (4) ...my health will improve. |  |  |
|  |  | (5) ...people will look at me askance. |  |  |
|  |  | (6) ...I am less prone to illnesses. |  |  |
|  |  | (7) ...my friends find it ridiculous. |  |  |
|  |  | (8) ...I reduce my chances of a heart attack. |  |  |
|  |  | (9) ...I am more attractive for others (whiter teeth, better skin, more pleasant smell of clothes). |  |  |
|  |  | (10) ...I feel more comfortable. |  |  |
|  |  | (11) ... my social life is being compromised (e.g. parties, in an association, with friends). |  |  |
|  |  | (12) ...my blood values improve (cholesterol). |  |  |
|  |  | (13) ...it makes it harder for me to relax. |  |  |
|  |  | (14) ...means less quality of life for me. |  |  |
|  |  | (15) …I feel more at ease mentally. |  |  |
|  |  | (16) ...I will gain weight. |  |  |
|  |  | (17) ...I am a good role model (e.g. for my partner or my children). |  |  |
|  |  | (18) ...other people value my willpower. |  |  |
| Action planning (F) | Action plans and coping plans for physical exercise [33] | (1) Do you already know which physical exercises you want do in the next 4 weeks? | Yes  No | 1 2 |
|  |  | (2) Which physical exercises do you plan to do in the next 4 weeks? | Activity: [50 characters]  (please enter here) |  |
|  |  | I already know for activity A… |  |  |
|  |  | (3) ...**when** I will carry it out. | Yes  No | 1 2 |
|  |  | (4) ...**where** I will carry it out. |  |  |
|  |  | (5) ...**how** I will get there. |  |  |
|  |  | (6) ...**how often** I will carry it out. |  |  |
|  |  | (7) ...**with whom** I will carry it out. |  |  |
|  |  | (8) Which sports activities do you plan to carry out in the next four weeks? | Activity B: [50 characters]  (please enter here) |  |
|  |  | I already know for activity B... |  |  |
|  |  | (9) ...**when** I will carry it out. | Yes No | 1 2 |
|  |  | (10) ..**.where** I will carry it out |  |  |
|  |  | (11) ...**how** I will get there. |  |  |
|  |  | (12) ...**how often** I carry it out. |  |  |
|  |  | (13) ...with **whom** I will carry it out. |  |  |
| Risk perception (S) | Risk perception [34] - adapted | Do you think that the following things can happen to you if you keep smoking? | “Scale 0-6“  0 =very unlikely  6 = very likely“ |  |
|  |  | (1) Lung cancer |  |  |
|  |  | (2) Other lung disease |  |  |
|  |  | (3) Heart disease |  |  |
|  |  | (4) become dependent |  |  |
|  |  | (5) Premature death |  |  |
| Perceived goal attainment (W) | Self-developed | (1) To what extent have you succeeded in achieving your goal of a calorie-conscious or healthy diet? | More than 100 % of the goal achieved (= exceeded)  100% of the goal achieved  75% of the goal achieved  50 % of the goal achieved  25 % of the goal achieved  0 % of the goal achieved (unchanged)  Less than 0 % of the goal achieved (worse than before) | 7  6 5 4 3 2 1 |
|  |  | (2) How satisfied are you with what you have achieved? | Very satisfied  Satisfied  Neither satisfied nor dissatisfied  Dissatisfied  Extremely dissatisfied | 5 4 3 2 1 |
| Perceived goal attainment (F)  Perceived goal attainment (F) (Continuation) | Self-developed  Self-developed | (1) To what extent have you succeeded in achieving your goal of being regularly active in sports? | More than 100 % of the goal achieved (= exceeded)  100% of the goal achieved  75% of the goal achieved  50 % of the goal achieved  25 % of the goal achieved  0 % of the goal achieved (unchanged)  Less than 0 % of the goal achieved (worse than before) | 7  6 5 4 3 2 1 |
|  |  | (2) How satisfied are you with what you have achieved? | Very satisfied  Satisfied  Neither satisfied nor dissatisfied  Dissatisfied  Extremely dissatisfied | 5 4 3 2 1 |
| Perceived goal attainment (S) | Self-developed nt | (1) Have you achieved your goal to stop smoking? | Yes  Partially  No | 3 2 1 |
|  |  | (2) How satisfied are you with what you have achieved? | Very satisfied  Satisfied  Neither satisfied nor dissatisfied  Dissatisfied  Extremely dissatisfied | 5 4 3 2 1 |
| Eating habits (W,F)  Eating habits (W,F) (Continuation)  Eating habits (W,F) (Continuation) | German Eating Habits Questionnaire (FEG); self-developed , article in preparation [21]^*^  FEG; self-developed, article in preparation [21]^*^  FEG; self-developed , article in preparation [21]^*^ | The following is a general discussion about your diet.  What applies to you? |  |  |
|  |  | (1) I have a food intolerance. | Yes  No | 1 2 |
|  |  | (2) If so, which ones? | [150 characters] (please enter here) |  |
|  |  | (3) I eat a vegetarian diet. | Scale 1 to 5  1 = does not apply at all to 5 = applies exactly  Scale 1 to 5  1 = does not apply at all to 5 = applies exactly  Scale 1 to 5  1 = does not apply at all to 5 = applies exactly |  |
|  |  | (4) I eat a vegan diet. |  |  |
|  |  | (5) I eat a low fat diet. |  |  |
|  |  | (6) If I have eaten too much one day, I eat less the next day. |  |  |
|  |  | (7) I eat a lot of ready-made meals. |  |  |
|  |  | (8) I choose regularly the wholegrain variety, when I eat cereal products (bread, noodles, rice, flour). |  |  |
|  |  | (9) I prefer organic food. |  |  |
|  |  | (10) I use salt sparingly. |  |  |
|  |  | (11) I consciously hold back on eating so as not to gain weight. |  |  |
|  |  | (12) I take my time to eat. |  |  |
|  |  | (13) I eat everything I want and when I want it. |  |  |
|  |  | (14) I choose products of regional agriculture. |  |  |
|  |  | (15) My diet is wide and varied. |  |  |
|  |  | (16) I don’t eat between meals as I am worried about my weight. |  |  |
|  |  | (17) I eat a lot of fast food. |  |  |
|  |  | (18) I don’t eat late at night as I pay attention to my weight. |  |  |
|  |  | (19) I eat a lot of fresh fruit. |  |  |
|  |  | (20) I cook my own meals. |  |  |
|  |  | (21) I eat a lot of fresh vegetables. |  |  |
|  |  | (22) I often decline dishes or drinks as I am worried about my weight. |  |  |
|  |  | (23) I try to eat low-calorie food. |  |  |
|  |  | (24) I often nibble savory biscuits/peanuts/chips. |  |  |
|  |  | (25) I prefer the lower fat version for certain food. |  |  |
|  |  | (26) I eat quite a lot for my standards. |  |  |
|  |  | (27) I eat less sugar. |  |  |
|  |  | (28) When I have gained weight, I eat less than usual. |  |  |
|  |  | (29) I prefer seasonal groceries. |  |  |
|  |  | (30) I eat a lot of meat or sausages. |  |  |
|  |  | (31) In the evening, I drink more than a glass of alcohol. |  |  |
|  |  | (32) I intentionally eat less in order not to gain weight. |  |  |
| General state of health (W,F,S) | BRAHMS [26] - adapted | When I compare myself to other people of my age and sex, my current state of health is... | Scale 0-10  0=considerably worse to 10=considerably better |  |
| HG = Health goals, Health goals: W=*Losing and Maintaining Weight*; F=*Increasing Fitness*, S=*Smoking Cessation* | | | | |
