# Supplementary material for: Effectiveness of an interactive web-based health program for adults: a study protocol for three concurrent controlled-randomized trials (EVA-TK-Coach)
Source: Trials. 2021 Aug 10;22:526. doi: 10.1186/s13063-021-05470-8 (PMC8353439; doi:10.1186/s13063-021-05470-8)
Supplement: Supplementary file 5 — Additional file 5:. [file 13063_2021_5470_MOESM5_ESM.docx]

| **Variables / Constructs (HG)** | | **Source / Origin** | **Questions** | **Answer Options** |  |
| --- | --- | --- | --- | --- | --- |
| *Confounder* | | | | | |
| Health impairments (W,F,S)  Health impairments (W,F,S) (Continuation)  Health impairments (W,F,S) (Continuation) | | KOMO  Comorbidity Score (KoMo) [39]  KoMo [39]  KoMo [39] | Do you have the following health issue? |  |  |
|  |  |  | (1) High blood pressure |  |  |
|  |  |  | (2) If “yes“: Are you limited in your daily activities because of this particular disease? | No  Yes, a little  Yes, fairly  Yes, severely | 1 2 3 4 |
|  |  |  | (3) Heart attack (in the past) |  |  |
|  |  |  | (4) If “yes“: Are you limited in your daily activities because of this particular disease? | No  Yes, a little  Yes, fairly  Yes, severely | 1 2 3 4 |
|  |  |  | (5) Circulatory disorder/ Vascular diseases |  |  |
|  |  |  | (6) If “yes“: Are you limited in your daily activities because of this particular disease? | No  Yes, a little  Yes, fairly  Yes, severely | 1 2 3 4 |
|  |  |  | (7) Disease of the respiratory organs |  |  |
|  |  |  | (8) If “yes“: Are you limited in your daily activities because of this particular disease? | No  Yes, a little  Yes, fairly  Yes, severely | 1 2 3 4 |
|  |  |  | (9) Gastrointestinal disorder |  |  |
|  |  |  | (10) If “yes“: Are you limited in your daily activities because of this particular disease? | No  Yes, a little  Yes, fairly  Yes, severely | 1 2 3 4 |
|  |  |  | (11) Disease of the liver/gall |  |  |
|  |  |  | (12) If “yes“: Are you limited in your daily activities because of this particular disease? | No  Yes, a little  Yes, fairly  Yes, severely | 1 2 3 4 |
|  |  |  | (13) Kidney disease |  |  |
|  |  |  | (14) If “yes“: Are you limited in your daily activities because of this particular disease? | No  Yes, a little  Yes, fairly  Yes, severely | 1 2 3 4 |
|  |  |  | (15) Diabetes |  |  |
|  |  |  | (16) If “yes“: Are you limited in your daily activities because of this particular disease? | No  Yes, a little  Yes, fairly  Yes, severely | 1 2 3 4 |
|  |  |  | (17) Diseases of musculo-skeletal system (chronic back pain, joint disease) |  |  |
|  |  |  | (18) If “yes“: Are you limited in your daily activities because of this particular disease? | No  Yes, a little  Yes, fairly  Yes, severely | 1 2 3 4 |
|  |  |  | (19) Neurological disease (stroke, multiple sclerosis) |  |  |
|  |  |  | (20) If “yes“: Are you limited in your daily activities because of this particular disease? | No  Yes, a little  Yes, fairly  Yes, severely | 1 2 3 4 |
|  |  |  | (21) Cancer |  |  |
|  |  |  | (22) If “yes“: Are you limited in your daily activities because of this particular disease? | No  Yes, a little  Yes, fairly  Yes, severely | 1 2 3 4 |
|  |  |  | (23) Depression, Anxiety |  |  |
|  |  |  | (24) If “yes“: Are you limited in your daily activities because of this particular disease? | No  Yes, a little  Yes, fairly  Yes, severely | 1 2 3 4 |
| *Social support* | |  |  |  |  |
| Social support (W)  Social support (W) (Continuation) | | Support for Weight Management [41] – adapted  Support for Weight Management [41] - adapted | Please answer the following questions even if you don’t eat calorie-conscious or healthy (yet)  People who are close to me... |  |  |
|  |  |  | (1) ...believe that I am able to eat a calorie-conscious or healthy diet and that I can reduce my weight. | (Hardly) ever  Sometimes  Often  (Almost) always  (Hardly) ever  Sometimes  Often  (Almost) always | 1 2 3 4  1 2 3 4 |
|  |  |  | (2) ...tell me that I won`t be able to reduce my weight long turn. |  |  |
|  |  |  | (3) ...prepare calorie-conscious or healthy meals with me. |  |  |
|  |  |  | (4) ...pay me compliments on my change in eating patterns (e.g. “Keep it up“, “We are proud of you“) |  |  |
|  |  |  | (5) ...help me to have less food rich in calories or unhealthy food at home. |  |  |
|  |  |  | (6) ...listen to me if I try to solve difficulties in relation to weight loss. |  |  |
|  |  |  | (7) ...try to understand the challenges of my weight loss without judging me. |  |  |
|  |  |  | (8) ...ask me how they can best support me to lose weight. |  |  |
|  |  |  | (9) ... remind me of the important reasons why I want to lose weight when I'm unmotivated. |  |  |
|  |  |  | (10) ...encourage me to eat high-calorie or unhealthy food by saying things like "An exception won't make a difference" or "Don't worry about your weight". |  |  |
|  |  |  | (11) ...react angrily when I encourage them to eat calorie-conscious or healthy food. |  |  |
|  |  |  | (12) ...offer me dishes that I try to avoid. |  |  |
|  |  |  | (13) ...eat high-calorie or unhealthy food in front of me. |  |  |
|  |  |  | (14) ...refuse to eat calorie-conscious or healthy food with me. |  |  |
| Social support (F)  Social support (F) (Continuation) | | Social support [42] (in prep.)  Social support [42] (in prep.) | Please answer the following questions even if you do not (yet) do any sports yourself.  People who are close to me... |  |  |
|  |  |  | (1) ...do sports with me or would do sports with me. | (Hardly) ever  Sometimes  Often  (Almost) always  (Hardly) ever  Sometimes  Often  (Almost) always | 1 2 3 4  1 2 3 4 |
|  |  |  | (2) ...ask me to start or continue with sports. |  |  |
|  |  |  | (3) ...are not considerate with my sports activities. |  |  |
|  |  |  | (4) ...support me practically (e.g. in the household), so that I can / could practice my sports activities. |  |  |
|  |  |  | (5) ...inquire about my sporting activities. |  |  |
|  |  |  | (6) ...make disparaging remarks when I do sports / would do sports. |  |  |
|  |  |  | (7) ...encourage me to do sports. |  |  |
|  |  |  | (8) …remind me to do sports regularly. |  |  |
|  |  |  | (9) ...keep me from doing sports. |  |  |
|  |  |  | (10) ...are offering to do sports with me. |  |  |
|  |  |  | (11) ...complain about the time I spend / would spend doing sports. |  |  |
|  |  |  | (12) ...think it would be good if I did sports / would do sports. |  |  |
| Social support (S)  Social support (S) (Continuation) | | Partner Interaction Questionnaire (PIQ-20) [43] – adapted  PIQ-20 [43] - adapted | (1) Does your partner smoke? | Yes  No  I don’t have a partner | 1 2 3 |
|  |  |  | (2) How many people who are close to you smoke? | Nobody  Less than half  About half  More than half of the people  Everyone | 1 2 3 4 5 |
|  |  |  | Please answer the following questions even if you have not stopped smoking (yet).  People who are close to me ... |  |  |
|  |  |  | (3) ...ask me to stop smoking. | (almost) never  Sometimes  Often  (almost) always | 1 2 3 4 |
|  |  |  | (4) ...compliment me on not smoking. |  |  |
|  |  |  | (5) ... tell me not to smoke a cigarette. |  |  |
|  |  |  | (6) ... help me to think about substitutes for cigarettes. |  |  |
|  |  |  | (7) ...are expressing confidence that I can/will make it/endure. |  |  |
|  |  |  | (8) ... smoke in my immediate presence. |  |  |
|  |  |  | (9) ...react angrily when I encourage them to stop smoking. |  |  |
|  |  |  | (10) ...make derogatory remarks about me not smoking or not smoking anymore. |  |  |
|  |  |  | (11) ... make remarks that smoking is a bad habit. |  |  |
|  |  |  | (12) ... forbid me to smoke inside the house/flat. |  |  |
|  |  |  | (13) ... offer me cigarettes. |  |  |
|  |  |  | (14) ...don't care if I don't smoke or wouldn't smoke anymore. |  |  |
|  |  |  | (15) ... help me to calm down when I feel stressed or irritable. |  |  |
|  |  |  | (16) ... tell me that my smoking bothers them. |  |  |
|  |  |  | (17) ... ...do something with me to keep me from smoking (e.g. go for a walk) |  |  |
| Evaluation of the program | | | | | |
| Use and evaluation of the online programs (W,F,S)  Use and evaluation of the online programs (W,F,S) (Continuation)  Use and evaluation of the online programs (W,F,S) (Continuation) | | Self-developed  Self-developed  Self-developed | (1) How long have you used the online health program?  The program... | ....I have used it so far and will continue to use it.  ...I have used it so far and now I stop using it.  I haven’t used it for quite some time.  I haven’t used it at all. | 4  3 2 1 |
|  |  |  | Filter questions |  |  |
|  |  |  | (2) If 4 “I have used it so far and will continue using it“:  How many weeks have you used the program so far? | [two-digit number field] weeks |  |
|  |  |  | (2) If 3 “I have used it so far and now I will finish it“:  How many weeks have you used the program? | [two-digit number field] weeks |  |
|  |  |  | (2) If 2 “I haven’t used it for quite a while“: For how many weeks have you used the program in total? | [two-digit number field] weeks |  |
|  |  |  | (3) With which device did you **mainly** access and use the online health program of the health survey? | Smartphone  Tablet  Laptop / notebook  Booth computer / computer  Other | 1 2 3 4 5 |
|  |  |  | (4) Did technical problems occur while you used the online health program? | No  Yes | 2 1 |
|  |  |  | Filter question: |  |  |
|  |  |  | (5) If “Yes“:  Which problems did you have?  *(Key words sufficient)* | [free text 250 characters] |  |
|  |  |  | (6) Have you used the telephone hotline for your health program for technical or content-related questions? | No  Yes | 2 1 |
|  |  |  | Filter question: |  |  |
|  |  |  | (7) If “yes“:  Did you get any help with your request? | Yes  Partly  No | 1 2 3 |
|  |  |  | (8) How well did you find your way around the online health program pages? | Very bad  Bad  Average  Good  Very good | 1 2 3 4 5 |
|  |  |  | (9) Have you looked at the content of the online health program in detail? | no  rather no  partly  rather yes  yes | 1 2 3 4 5 |
|  |  |  | The content of the online health program was... |  |  |
|  |  |  | (10) ...interesting. | strongly disagree  disagree  somewhat disagree  somewhat agree  agree  strongly agree | 1 2 3 4 5 6 |
|  |  |  | (11) ... easy to understand. |  |  |
|  |  |  | (12)...attractively designed. |  |  |
|  |  |  | The online health program has... |  |  |
|  |  |  | (13)…provided me useful suggestions | strongly disagree  disagree  somewhat disagree  somewhat agree  agree  strongly agree | 1 2 3 4 5 6 |
|  |  |  | (14) ...was fun. |  |  |
|  |  |  | (15) ...has shown me what I can do to achieve my health goal. |  |  |
|  |  |  | (16) ...contributed to the fact that I will continue to pursue my health goal in the future. |  |  |
|  |  |  | (17) Overall, which grade would you give the online health program? | 1 (very good)  2 (good)  3 (average)  4 (sufficient)  5 (poor)  6 (deficient) | 1 2 3 4 5 6 |
|  |  |  | (18) Would you recommend the online health program? | No  Maybe  Yes | 1 2 3 |
|  |  |  | (19) Is there anything else that you would like to tell us about the health program? | [Free text 250 characters] |  |
| Premature program termination (W,F,S)  Premature program termination (W,F,S) (Contiunation) | | Self-developed  Self-developed | (1) Why did you terminate the online health program prematurely or not use it at all?  (Multiple answers possible) | Personal reasons (e.g. illness, professional or family-related reasons, extended absence from home, I no longer pursue the health goal at the moment)  Reasons that refer to the online health program? (e.g. Content of the program, handling of the program, excessive demands, low demands)  I had technical issues (no stable or low internet connection, issues with my computer, on-screen display illustration) | 1    2   3 |
|  |  |  | Filter question |  |  |
|  |  |  | If figure 1  (2) What personal reasons led you to terminate the online health program prematurely or not use it at all? | Illness  Professional or family-related reasons  Extended absence from home  I no longer pursue the health goal at the moment  Other: [250 characters] | 1 2 3  4  5 |
|  |  |  | If figure 2  (3) Which program-related reasons led you to terminate the online health program prematurely or not use it at all?  (*Multiple answers possible*) | The content of the program did not meet my expectations  The program was too time consuming  The program does not work for me, has no effect on me.  I did not get any new suggestions.  I felt overwhelmed.  I felt unchallenged.  The program was too complicated.  Other: [250 characters] | 1  2 3  4 5 6 7 8 |
|  |  |  | If figure 3  (4) Which technical issues led you to terminate the online health program prematurely or not used it at all?  (*Keywords suffice*) | [free text 250 characters] |  |
|  |  |  | (5) What would you have wished for the online-program in order to participate longer? | [free text 250 characters] |  |
| Text box for additional comments | Self-developed | | If you would like to tell us something important or have any comments, please enter them in the comment field: | Comments/Suggestions [300 characters] |  |
| HG = Health goals: W=*Losing and Maintaining Weight*; F=*Increasing Fitness*, S=*Smoking Cessation* | | | | | |
