# Supplementary material for: Effectiveness of an interactive web-based health program for adults: a study protocol for three concurrent controlled-randomized trials (EVA-TK-Coach)
Source: Trials. 2021 Aug 10;22:526. doi: 10.1186/s13063-021-05470-8 (PMC8353439; doi:10.1186/s13063-021-05470-8)
Supplement: Supplementary file 6 — Additional file 6:. [file 13063_2021_5470_MOESM6_ESM.pdf]

Research Project
Evaluation of the *Techniker health insurance fund's* web-based health programs (Fitness)

STUDY INFORMATION

October 2019

Dear Prospective Students,

Techniker health insurance fund offers web-based health programs whose quality and benefits are investigated by independent scientific institutes. To conduct the present evaluation, the *Techniker* has commissioned the University Medical Center Freiburg (represented by the Section of Health Care Research and Rehabilitation Research, SEVERA) and the University of Freiburg (represented by the Department of Sport and Sport Science, IfSS). Vilua Healthcare GmbH is responsible for the technical implementation.

We would like to ask you to participate in the survey. Your participation is, of course, voluntary. In case of non-participation, you will not experience any disadvantages. If you do not wish to participate in the survey, you can still participate in the *Techniker's* online health program (www.tk.de), but under limited conditions. Before you decide, we would first like to explain the process, content and objectives of the project.

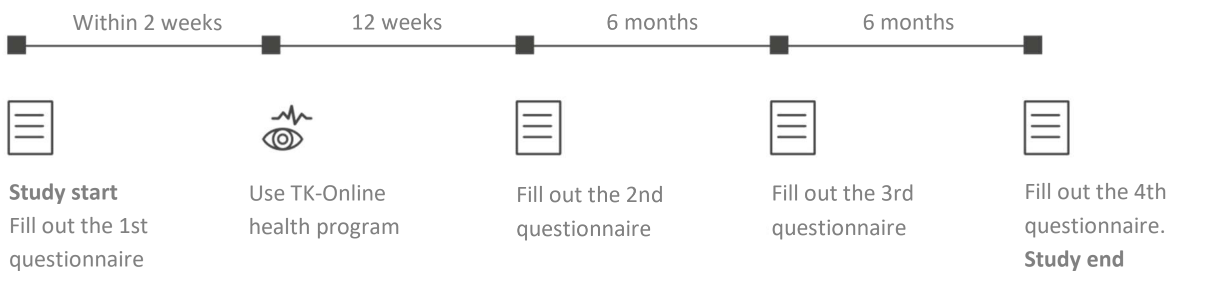

Project description

The project aims to investigate the use and effectiveness of differently presented online programs of the *Techniker health insurance fund* for various health goals. The present sub-study of the project refers to the health goal: increasing physical activity and fitness, in short "fitness". For this purpose, a modern, interactive online program (intervention group) is to be compared with a lean, classically prepared (non-interactive) program (control group). The online programs are recommendations for action and include scientifically based guidelines which support participants in achieving their personal goals in respect of their physical fitness.

To evaluate the use and effectiveness of the two programs, data from individuals using the interactive online program (intervention group) must be compared to those using the traditional online program (control group).

Inclusion criteria
You can participate if you have reached the age of 18 and are not currently pregnant. Participation is also possible if you are not insured with *Techniker health insurance fund*.

Enrollment
Your participation in the study begins with your confirmation of the declaration of consent on the following page. At the bottom of this page and on the following page, you also have the option of saving and printing this study information and the declaration of consent so that you can read the study information at any time and know what you have consented to. The study information and your declaration of consent will also be automatically saved in your personal profile of the health program.

Information of participants
After registering for the study, you can use the online health program provided for the entire duration of the study (expected end, Fall 2021). To assess use and effectiveness, you will receive an online questionnaire prior to initial use, after completion of the recommended use period, i.e. after 12 weeks, as well as after 6 months and finally after 12 months, respectively. In these surveys, you will be asked to evaluate the online program used and to answer questions about health-related attitudes, motives, and behaviors, particularly in relation to the goal of the online program (fitness). In order to evaluate the use and effectiveness of the online program (fitness), data on your usage behavior (user data) will also be collected and linked to your answers given in the online questionnaires. Usage behavior data includes, for example, the frequency and duration of use, the specified number of behavioral goals, and the number of logged activities.

End of participation/withdrawal
Participation in the study is voluntary. You can withdraw your participation at any time, even if consent has already been given, without giving your reasons. If you do not wish to participate, you do not need to confirm your consent. The legal basis for data processing is your consent to provide personal data. You are under no legal or contractual obligation to do so. Not providing it would result in less data being available for scientific purposes, but no disadvantages for you personally. If you wish to terminate your participation in the study, a corresponding function is available to you in the personal profile of your health program ("Terminate study participation" button). In addition to this digital termination, you can also contact Vilua Healthcare GmbH by phone (Tel.: 030 40 740 655) or by e-mail (info@tk-studie.vilua.de), stating your user name, to terminate your participation. Vilua Healthcare GmbH is responsible for the technical implementation of the health program and the management of the declarations of consent and personal data.

In case of a subsequent withdrawal from participation, all the personal data already provided (e.g. e-mail address) and your declaration of consent will be deleted. However, the retroactive deletion of your research data (questionnaire data and user data) is not possible. If you withdraw your participation, your previous research data will still be used in anonymized form for scientific research of the program. From the time of your withdrawal, no further questionnaire data will be collected and no further (user) data will be stored for study purposes.

However, the online health program will still be available to you after revocation of study participation until the end of the study period (presumably, Fall 2021). The data stored in this context will be used exclusively for your personal use of the online health program. Neither participation, nor non-participation, in the study will result in any disadvantages for you.

Participation in other research projects

During your participation in this study, you may not participate in any other research project on dietary and/or physical activity behaviors. Participation in other research projects may affect the results of this study.

Information on data protection

The legal provisions of data protection are applied. Your personal data will only be stored by Vilua Healthcare GmbH on password-protected, secure systems and will be kept strictly separate from your information in the questionnaires. In order to be able to combine your information in the questionnaires with the user data (research data), a unique pseudonym is created for you in form of a consecutive number. Your research data, pseudonymized in this way, does not allow any conclusions to be drawn about you personally, and are only transmitted to the scientific institutes involved (i.e. SEVERA and IfSS). Your data will only be processed within the scope of the consent given (legal basis) and only for the purpose of the described research project. Your data may be used in a summarized form over all the participants of your group (intervention or control group), either in complete form or in excerpts, in research reports, training courses or further publications. In case of publication, however, no personal data will be disclosed.

Should it become necessary for additional persons to have access to the data (e.g., due to the expansion of the project team), they are also obligated to treat the data as strictly confidential.

The responsible party within the meaning of Article 13 (1a) of the EU General Data Protection Regulation (EU GDPR) is the University Medical Center Freiburg (Universitätsklinikum Freiburg, Breisacher Straße 153, 79110 Freiburg; www.uniklinik-freiburg.de). If you have any data protection-related questions about the study, you can contact the data protection officer of the University Medical Center Freiburg in addition to the contacts listed below (datenschutz@uniklinik-freiburg.de). In addition, you can contact the State Commissioner for Data Protection and Freedom of Information of Baden-Württemberg (Tel.: 0711 61 5541-0; https://www.baden-wuerttemberg.datenschutz.de) for all data protection-related matters (e.g., complaints).

In principle, all the statutory data subject rights apply to you. This means:

- that you have a right to inquire which of your data has been processed (right to information)
- that you can have data blocked or deleted under certain conditions (restriction of processing or deletion)
- that you can have data corrected (rectification)
- that those responsible must inform all the possible recipients about corrections, deletions or restrictions
- that you can have your data transferred to yourself or others (data portability)

If you wish to exercise any of these rights, please get in touch with the Vilua Healthcare GmbH contact person listed below. In the course of this contact with Vilua, an identification process

takes place in order to safeguard your data subject rights, during which personal information is requested (user name in the healthcare program, age, gender, e-mail address, the first two digits of the postal code).

Data exchange

By accepting the declaration of consent, you expressly give your permission for your data to be forwarded in pseudonymized form to the Section of Health Care Research and Rehabilitation Research and to the Department of Sport and Sport Science of the University of Freiburg. You can object to this data transfer at any time without stating your reasons.

The use of the health program is recorded with the help of web analytics software. This records, for example, which program elements (e.g. videos) are called up and how often, which behavioral goals and activities are selected, and how often, and for how long, the program is used. The analysis of these data is used to improve the program and feeds into the evaluation of the program's effectiveness. The Online Health Program also uses session cookies to authenticate you as a user during a session. In the program itself, you will once again be made aware of this by means of a banner.

Data deletion

Your consent and personal data will be digitally archived at Vilua Healthcare GmbH's hosting service provider, Claranet GmbH, in an access-restricted and strongly encrypted file storage for another 36 months after the expected completion of the entire research project in 2022. The archiving is done in order to prove the proper execution of the research project in case of possible controls, e.g. by scientific advisory boards or data protection officers. Each access is controlled by the project responsible persons. After expiration of the mentioned period, all personal data will be deleted. All research data (questionnaires and user data) will be archived in accordance with the guidelines of the German Research Foundation on Good Scientific Practice for ten years after the end of the project in the Section of Health Care Research and Rehabilitation Research and in the Department of Sport and Sport Science on a secure, password-protected project drive, to which only authorized project staff have access, and will be destroyed after this period.

Questions

If you have any questions about the procedure and contents of the study, you can contact the persons responsible for the research project, the Section of Health Care Research and Rehabilitation Research of the University Medical Center Freiburg (SEVERA). In order to protect your anonymity vis-à-vis the staff in case of questions regarding the content of the study, please make sure not to provide any information that could be used to infer your identity or your participation in the study when contacting SEVERA. For questions regarding administrative processing (e.g., termination of your participation) or the contents of the health program, please contact Vilua Healthcare GmbH.

Responsible for the survey via online questionnaire

Section of Health Care Research and Rehabilitation Research (SEVERA) of the University Medical Center Freiburg, Hugstetter Str. 49, 79106 Freiburg.

Contact person	Ms. Iris Tinsel	(Tel: 0761/270-74420)
	Prof. Dr. Erik Farin-Glattacker	(Tel: 0761/270-74430)
	E-mail address	bemb.tk-studie@uniklinik-freiburg.de

Contact person company Vilua Healthcare GmbH

Vilua Healthcare GmbH, Wohlrabendamm 32, 13629 Berlin, Tel.: 030 40 740 655, E-mail address: info@tk-studie.vilua.de

The division of study-related and administrative processing among different institutions serves to protect you personally and to strictly separate personal data from research data.

Thank you very much for your participation!

Research Project
Evaluation of the *Techniker health insurance fund*'s web-based health programs

DECLARATION OF CONSENT

1. I hereby confirm that I have received an information letter explaining the content and objectives, as well as data protection issues of the study. I have had sufficient time to think about it and all my questions have been answered satisfactorily. The implementation of the project is coordinated and supervised by the Section of Health Care Research and Rehabilitation Research (SEVERA) of the University Medical Center Freiburg.

2. I have been informed that I have the right to revoke this consent, in whole or in part, at any time. I do not need to state my reasons, nor do I have to continue participating in the study. In this case, all the personal data already provided (e.g. my e-mail address) and my declaration of consent will be deleted. A retroactive deletion of my research data (i.e. information in the questionnaires and user data) is not possible. In case of termination of my participation, my research data will be included in the scientific analyses in anonymized form. From the time of my revocation, no further questionnaire data will be collected from me, and no further (user) data will be stored for study purposes. However, the online health program will still be available to me after revocation of study participation until the end of the study period (presumably Fall 2021). The data stored in this context will be used exclusively for my personal use of the online health program. There will be no disadvantages for me, neither from participation, nor non-participation in the study.

3. I agree that my data will be forwarded and evaluated as described in the study information. The analyses of the pseudonymized data for the evaluation of the health program is carried out by the Section of Health Care Research and Rehabilitation Research of the University Medical Center Freiburg and the Department of Sport and Sport Science of the University of Freiburg.

4. I have been informed that I can object to the disclosure of data at any time and without stating my reasons. However, this will result in my not being able to continue participating in the research study.

5. Personal data or other information that allow conclusions to be drawn about my person will not be passed on to third parties or published. All the personal data will be deleted 36 months after completion of the evaluation project. The persons responsible will ensure that all the data collected is treated in strict confidence and used exclusively within the scope of the purpose of this study. The results of the study will be presented exclusively in anonymized form.

Under the conditions listed here, and in the study information, I agree to participate in the research study. My consent, as well as the study information, will be automatically stored in the personal area of my health program and can be viewed by me there at any time and revoked if necessary.

Reject Confirm Print
